# *Limosilactobacillus reuteri* and caffeoylquinic acid synergistically promote adipose browning and ameliorate obesity-associated disorders

**DOI:** 10.1186/s40168-022-01430-9

**Published:** 2022-12-15

**Authors:** Yameng Liu, Xianchun Zhong, Suqin Lin, Hualing Xu, Xinyu Liang, Yibin Wang, Jingyi Xu, Kanglong Wang, Xiaozhen Guo, Jiawen Wang, Minjun Yu, Cuina Li, Cen Xie

**Affiliations:** 1 State Key Laboratory of Drug Research, Shanghai Institute of Materia Medica, Chinese Academy of Sciences, Shanghai, 201203 People’s Republic of China; 2 School of Life Sciences, Shanghai University, Shanghai, 200444 People’s Republic of China; 3 School of Chinese Materia Medica, Nanjing University of Chinese Medicine, Nanjing, 210023 People’s Republic of China; 4 University of Chinese Academy of Sciences, Beijing, 100049 People’s Republic of China; 5 Shanghai Key Laboratory of Regulatory Biology, Institute of Biomedical Sciences and School of Life Sciences, East China Normal University, Shanghai, 200241 People’s Republic of China

**Keywords:** Obesity, Prebiotic, Probiotics, Short-chain fatty acids, Propionate, Thermogenesis, Browning

## Abstract

**Objective:**

High intake of caffeoylquinic acid (CQA)-rich dietary supplements, such as green coffee bean extracts, offers health-promoting effects on maintaining metabolic homeostasis. Similar to many active herbal ingredients with high pharmacological activities but low bioavailability, CQA has been reported as a promising thermogenic agent with anti-obesity properties, which contrasts with its poor oral absorption. Intestinal tract is the first site of CQA exposure and gut microbes might react quickly to CQA. Thus, it is of interest to explore the role of gut microbiome and microbial metabolites in the beneficial effects of CQA on obesity-related disorders.

**Results:**

Oral CQA supplementation effectively enhanced energy expenditure by activating browning of adipose and thus ameliorated obesity-related metabolic dysfunctions in high fat diet-induced obese (DIO) mice. Here, 16S rRNA gene amplicon sequencing revealed that CQA treatment remodeled the gut microbiota to promote its anti-obesity actions, as confirmed by antibiotic treatment and fecal microbiota transplantation. CQA enriched the gut commensal species *Limosilactobacillus reuteri* (*L. reuteri*) and stimulated the production of short-chain fatty acids, especially propionate. Mono-colonization of *L. reuteri* or low-dose CQA treatment did not reduce adiposity in DIO mice, while their combination elicited an enhanced thermogenic response, indicating the synergistic effects of CQA and *L. reuteri* on obesity. Exogenous propionate supplementation mimicked the anti-obesity effects of CQA alone or when combined with *L. reuteri*, which was ablated by the monocarboxylate transporter (MCT) inhibitor 7ACC1 or MCT1 disruption in inguinal white adipose tissues to block propionate transport.

**Conclusions:**

Our data demonstrate a functional axis among *L. reuteri*, propionate, and beige fat tissue in the anti-obesity action of CQA through the regulation of thermogenesis. These findings provide mechanistic insights into the therapeutic use of herbal ingredients with poor bioavailability via their interaction with the gut microbiota.

Video Abstract

**Supplementary Information:**

The online version contains supplementary material available at 10.1186/s40168-022-01430-9.

## Introduction

Obesity has become a pandemic and is closely linked to many chronic metabolic diseases, including type 2 diabetes, fatty liver diseases, hyperlipidemia, cardiovascular diseases, and even cancer [1, 2]. Obesity is caused by the complex interaction between environmental and genetic factors that results in an imbalance between energy intake and expenditure, ultimately leading to excessive energy storage in white adipose tissue (WAT) [3]. Contrary to WAT, brown adipose tissue (BAT) dissipates energy for heat production through uncoupled respiration mediated by mitochondrial uncoupling protein 1 (UCP1) [4]. Beige adipose tissues are interspersed within the WAT and are also crucial for thermogenesis, especially in humans. Transplantation studies suggest that both BAT and inguinal WAT (iWAT, the major site of beige adipocytes) can increase whole-body energy expenditure and improve insulin sensitivity [5, 6]. Thus, stimulating adaptive thermogenesis by brown and beige adipocytes is essential to re-establish whole-body energy homeostasis and therefore holds the potential for counteracting the development of obesity. In recent years, significant advances have been achieved in discovering new browning agents for the treatment of obesity. However, these agents exert undesired off-target effects, which might result in a series of adverse reactions [7]. Therefore, it is necessary to identify a novel strategy to increase energy expenditure to alleviate obesity-associated metabolic dysfunctions.

The connection between gut microbial dysbiosis and the development of obesity has attracted increasing attention. Evidence from both clinical and animal studies has suggested that the gut microbiota is indispensable to maintaining host energy homeostasis by regulating thermogenic processes in WAT [8, 9]. Cold acclimation and intermittent fasting could promote beiging of WAT by shaping the gut microbiota and ameliorating metabolic syndrome [10, 11]. Thus, strategies that target the gut microbiota to increase adaptive thermogenesis have been found beneficial to obesity-related disorders, such as fecal microbiota transplantation (FMT) [12]. Previous studies have shown that modulation of gut microbial composition using prebiotics and probiotics could improve host metabolism and reduce obesity [8, 13]. Gut bacteria, such as *Akkermansia muciniphila* and *Enterococcus faecalis*, have been reported to promote browning of WAT [8, 14]. The beneficial metabolic effects of gut bacteria, such as those mentioned above, are largely dependent on the production of microbial-derived metabolites, including short-chain fatty acids (SCFAs), secondary bile acids (BAs), and branched-chain amino acids (BCAAs) [15]. Thus, targeting gut microbiome and their metabolism pathways in particular, might be considered a promising strategy for treating obesity.

Coffee is one of the most popular beverages worldwide. Roasted coffee is a common form of coffee. The beneficial effects of coffee are mainly attributed to its caffeine content [16], whereas green coffee bean extracts are considered a novel thermogenic dietary supplement in humans because they are rich in caffeoylquinic acid (CQA; 45–55% in green coffee bean extracts) [17]. The phenolic acid structure of CQA with high polarity leads to poor oral absorption and low bioavailability [18]. Therefore, it is difficult to interpret the underlying mechanisms of its pharmacological properties. Although some studies have discovered several mechanisms for its metabolic improvements, including anti-oxidation, inhibition of liver glycogen synthesis, and suppression of fat absorption by a large dosage of CQA, it contrasts to its low bioavailability and clinical evidence remains limited [19–21]. The intestinal tract is the first site of CQA exposure, and gut microbes react quickly when they contact CQA. Several studies have discovered that CQA is metabolized by the gut bacteria to caffeic acid, indicating an inherent interaction between CQA and the microbiome, and vice versa, CQA potentially modulates the composition and function of the gut microbiota [22–24].

Considering the potential interaction of gut microbiota and CQA, we hypothesize that the beneficial effects of CQA on obesity-associated metabolic dysfunctions are mainly mediated by the modulation of gut microbiota. To test this scenario, we administered high fat diet-induced obese (DIO) mice orally with CQA, and the contribution of gut microbiota in the beneficial effects of CQA on metabolic disorders was evaluated using antibiotics treatment, FMT, 16S rRNA sequencing, combined probiotics with CQA treatment, and metabolite replenishment. We demonstrate that the gut microbiota was involved in the anti-obesity action of CQA, and identified a gut commensal bacterium *Limosilactobacillus reuteri* (*L. reuteri*) and a gut microbiota-derived metabolite propionate, which specifically responded to CQA treatment to promote adipose browning and thereby energy expenditure. Our current findings suggest a novel synergistic “probiotics-prebiotics” therapeutic strategy for the treatment of obesity and its complications by targeting obesity-induced gut dysbiosis.

## Results

### CQA treatment protected mice from diet-induced obesity and promoted energy expenditure

**Fig. 1:**
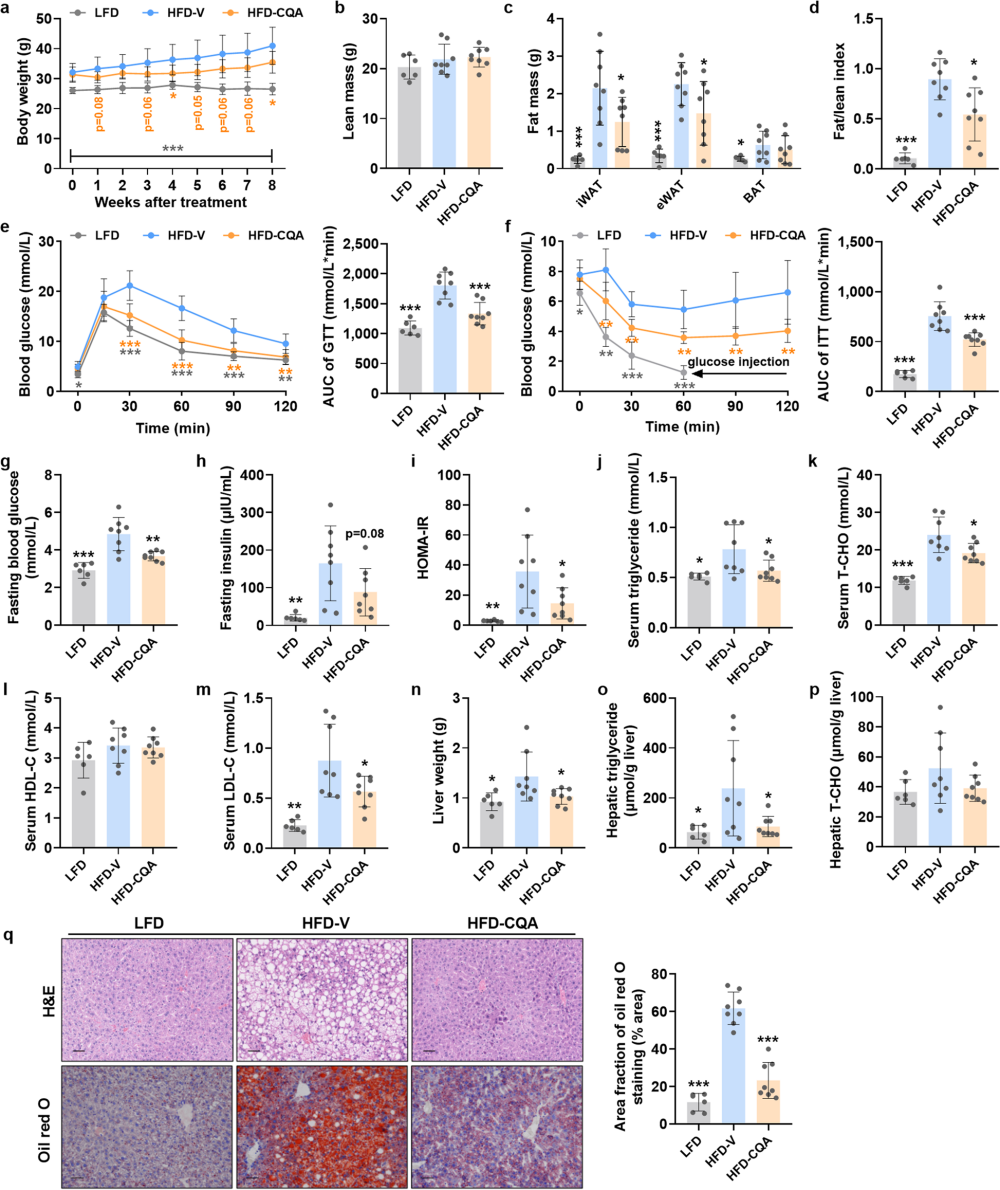
CQA attenuated high fat diet (HFD)-induced obesity (DIO) and metabolic disorders in mice. The mice were fed a HFD for 8 weeks and then orally treated with vehicle or CQA (150 mg/kg) daily for another 8 weeks. **a** Body weight. **b** Lean mass. **c** Fat mass. **d** Fat/lean index. **e** Glucose tolerance test (GTT) and area under the curve (AUC). **f** Insulin tolerance test (ITT) and AUC. **g** Fasting blood glucose (FBG). **h** Fasting insulin. **i** Insulin resistance (HOMA-IR) index. **j** Serum triglyceride. **k** Serum total cholesterol (T-CHO). **l** Serum high-density lipoprotein cholesterol (HDL-C). **m** Serum low-density lipoprotein cholesterol (LDL-C). **n** Liver weight. **o** Hepatic triglyceride. **p** Hepatic T-CHO. **q** Representative H&E staining (upper) and Oil red O staining (lower) of liver sections, scale bar: 50 μm. Lipids were stained positive (red color) with Oil red O and quantified by Image J software. LFD, *n* = 6/group. HFD-V and HFD-CQA, *n* = 8/group. Data are presented as mean ± SD. *p < 0.05; **p < 0.01; and ***p < 0.001 versus HFD-V

Consistent with previous reports [25], CQA treatment (HFD-CQA; 150 mg/kg daily) significantly prevented body weight gain in DIO mice (Fig. 1a). Compared with vehicle-treated (HFD-V) mice, the fat mass and the fat/lean index were substantially decreased in the HFD-CQA group without affecting lean mass (Fig. 1b–d). To clarify the role of CQA in obesity-related insulin resistance, a glucose-tolerance test (GTT) and insulin-tolerance test (ITT) were performed and revealed that CQA treatment improved glucose tolerance and increased insulin sensitivity (Fig. 1e, f). HFD-CQA mice displayed lower fasting blood glucose, insulin, and HOMA-IR index than HFD-V mice, which further confirmed the improvement of insulin resistance by CQA treatment (Fig. 1g–i). Moreover, the levels of serum triglyceride, total cholesterol (T-CHO), and low-density lipoprotein cholesterol (LDL-C), but not high-density lipoprotein cholesterol (HDL-C), were reduced in HFD-CQA mice than in HFD-V mice, indicating attenuated lipidemia (Fig. 1j–m). Decreased liver weight, hepatic triglyceride, and T-CHO (Fig. 1n–p), along with amelioration of fatty liver (Fig. 1q), suggested that hepatic steatosis was also alleviated by CQA. The data was consistent with reduced serum free-fatty acid (FFA) levels and decreased mRNA expression of lipogenesis-related genes in the liver (Fig. S1). The decreased palmitoleate to palmitate (16:1/16:0) and stearate to oleate (18:1/18:0) ratios, along with reduced mRNA expression of stearoyl-CoA desaturase (*Scd1*) in the liver, indicated improvement in obesity [26]. Taken together, these results revealed that oral administration of CQA prevents obesity and associated glucose and lipid dysregulation upon HFD consumption.

**Fig. 2:**
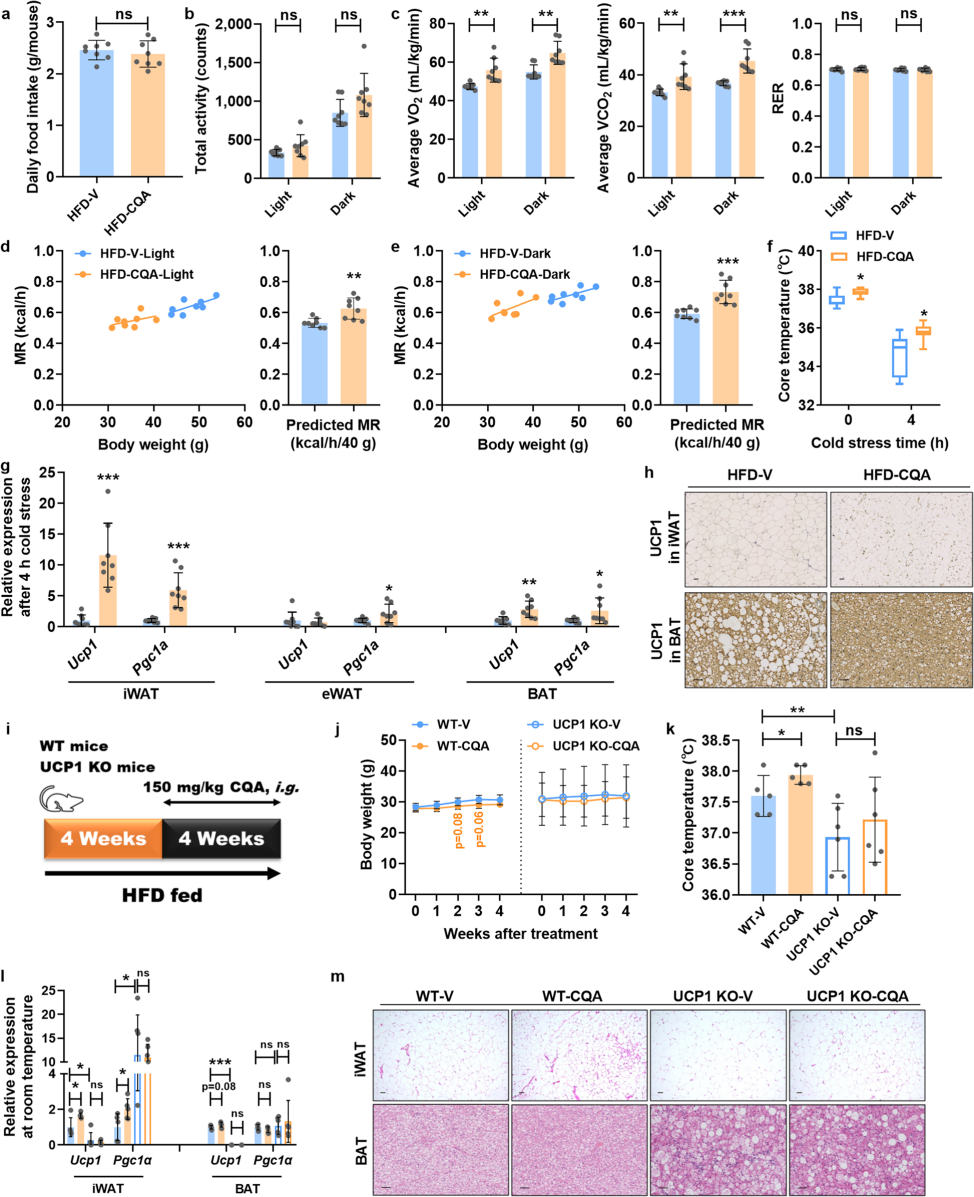
CQA enhances energy expenditure in obese mice. The mice were fed a HFD for 8 weeks and then orally treated with vehicle or CQA (150 mg/kg) daily for another 8 weeks. **a** Daily food intake. **b** Total activity. **c** VO_2_, VCO_2_, and respiratory exchange ratio (RER). **d**, **e** Predicted metabolic rate (MR) either in light (**d**) or dark (**e**). **f** Core temperature. **g** Relative mRNA expression of thermogenic genes in adipose tissues. **h** Representative UCP1 staining of iWAT sections (upper) and BAT sections (lower), scale bar: 50 μm. UCP1 knockout (UCP1 KO) mice and control wild type (WT) mice were fed a HFD for 4 weeks and then gavaged with vehicle or CQA (150 mg/kg) for 4 weeks. **i** Schematic diagram of treatment. **j** Body weight. **k** Core temperature at room temperature. **l** mRNA expression of thermogenic genes in iWAT and BAT. **m** Representative H&E staining of iWAT sections (upper) and BAT sections (lower), scale bar: 50 μm. **a**–**h** *n* = 8/group. **j**–**m** WT-V and WT-CQA, *n* = 5/group. UCP1 KO-V and UCP1 KO-CQA, *n* = 6/group. Data are presented as mean ± SD. *p < 0.05; **p < 0.01; and ***p < 0.001. ns means not statistically significant

Metabolic improvements in CQA-treated mice were not due to reduced caloric intake or induced activity (Fig. 2a, b), but were the consequence of enhanced energy metabolism. HFD-CQA mice maintained higher body temperature after cold exposure (Fig. 2f), indicating that CQA-treated mice maintained enhanced adaptive non-shivering thermogenesis by burning adipose tissue. To confirm the effect of CQA on energy expenditure, we subjected mice to indirect calorimetry analyses. As expected, HFD-CQA mice consumed more O_2_, released more CO_2_, and thereby substantially promoted energy expenditure (Fig. 2c). Moreover, we adjusted metabolic rates (MR) in these two groups that differed in body mass and composition [27] and observed that CQA treatment significantly increased predicted MR by falling on separate regression lines, indicating the mechanical effect caused by CQA on thermogenesis (Fig. 2d, e). Furthermore, the mRNA expression of thermogenesis-associated genes in adipose tissue was markedly induced after CQA treatment, specifically in iWAT and BAT, but not in visceral epididymal WAT (eWAT) (Fig. 2g). Consistently, CQA treatment increased the protein levels of UCP1 in both iWAT and BAT (Fig. 2h). Moreover, we further used UCP1 knockout (UCP1 KO) mice to evaluate the contribution of UCP1-dependent thermogenesis in the anti-obesity effects of CQA (Fig. 2i). The therapeutic effects of CQA, including body weight control and energy expenditure, were significantly improved in CQA-treated wild type (WT-CQA) mice compared with vehicle-treated wild type (WT-V) mice. However, UCP1 KO mice did not respond to the anti-obesity benefits of CQA administration (Fig. 2j-m). These results supported that CQA could stimulate UCP1-dependent thermogenesis to produce metabolic benefits.

### The anti-obesity effect of CQA depended on the gut microbiota

**Fig. 3:**
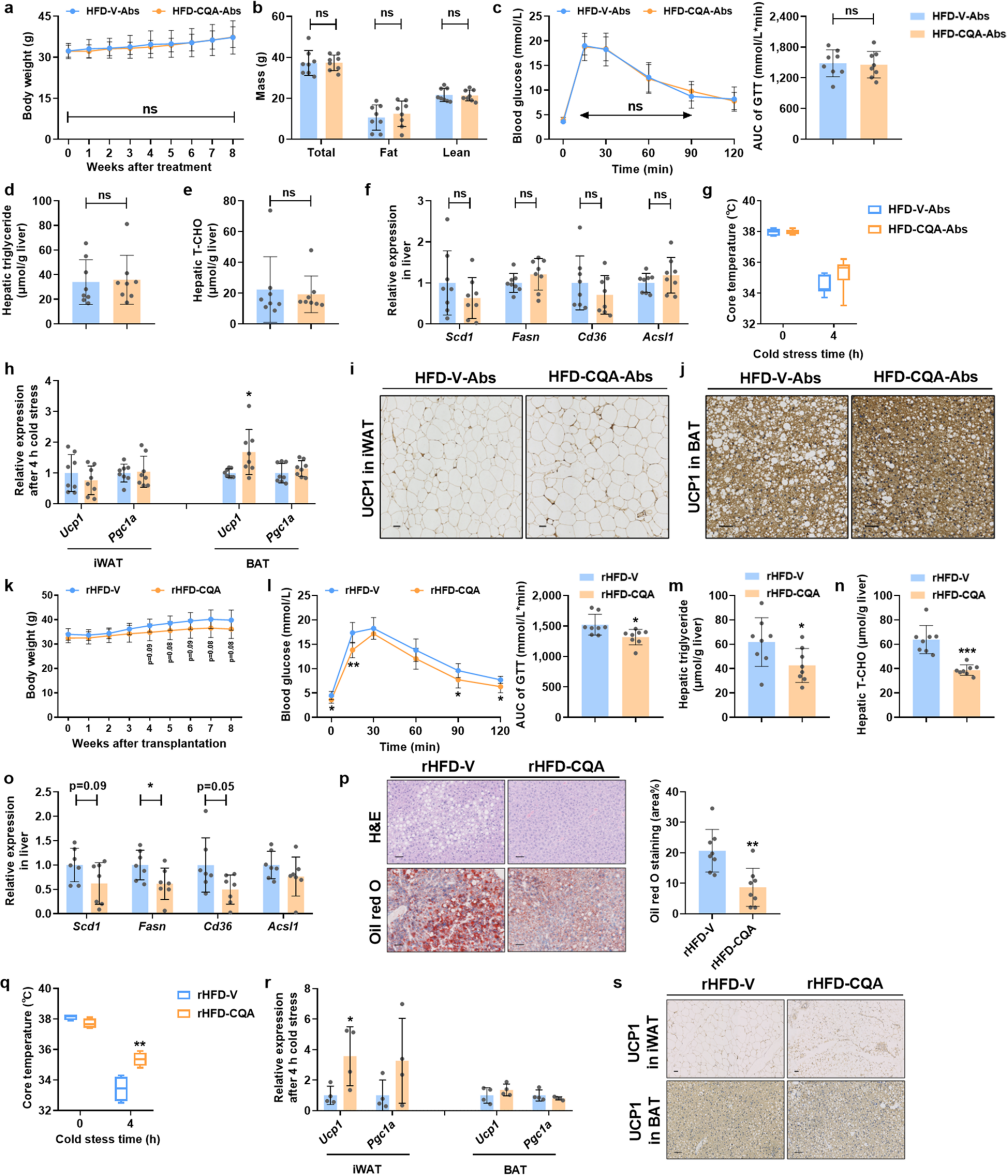
Gut microbiota mediate the therapeutic effects of CQA treatment. The mice were fed a HFD for 8 weeks and then gavaged with vehicle or CQA (150 mg/kg) together with an antibiotics cocktail in drinking water for 8 weeks. **a** Body weight of microbiota-reduced mice. **b** Fat mass. **c** GTT and AUC. **d** Hepatic triglyceride. **e** Hepatic T-CHO. **f** Hepatic mRNA expression of lipid synthesis related genes. **g** Core temperature. **h** Relative mRNA expression of thermogenic genes in iWAT and BAT. **i**–**j** Representative UCP1 staining of iWAT sections (**i**) and BAT sections (**j**), scale bar: 50 μm. The mice were fed a HFD for 8 weeks and then treated twice per week as recipient mice with fecal microbiota from vehicle-treated or CQA-treated DIO mice for another 8 weeks. **k** Body weight of fecal microbiota transplantation (FMT) mice. (l) GTT and AUC. **m** Hepatic triglyceride. **n** Hepatic T-CHO. **o** Hepatic mRNA expression of lipid synthesis-related genes. **p** Representative H&E staining (upper) and Oil red O staining (lower) of liver sections, scale bar: 50 μm. Lipids were stained positive (red color) with Oil red O, and quantified by Image J software. **q** Core temperature in FMT mice. **r** Relative mRNA expression of thermogenic genes in iWAT and BAT of FMT mice. **s** Representative UCP1 staining of iWAT sections (upper) and BAT sections (lower), scale bar: 50 μm. **a**–**p** *n* = 8/group. **q**–**s** *n* = 4/group. Data are presented as mean ± SD. *p < 0.05; **p < 0.01; and ***p < 0.001. ns means not statistically significant

CQA is a polar compound with poor bioavailability. Coincident with the previous finding that CQA can be hydrolyzed to caffeic acid (CA) by human gut bacteria (Fig. S2a) [28], we found that the serum levels of CQA were extremely low relative to its high dose (150 mg/kg). However, CA was unexpectedly almost undetectable in the serum of HFD-CQA mice (Fig. S2b). Therefore, it is reasonable to assume that CQA may indirectly influence thermogenesis by interacting with the gut microbiota. To identify whether the gut microbiota participates in the anti-obesity effect of CQA, we exposed DIO mice to an antibiotics cocktail via drinking water to reduce the majority of gut microbiota, which was supported by the amplicon sequencing data (Fig. S3a, b). As expected, the therapeutic effects of CQA disappeared after antibiotics intervention (Fig. 3a–f, Fig. S3c-h). It should be noted that CQA-treated microbiota-reduced (HFD-CQA-Abs) mice still maintained a relatively higher body temperature without statistical significance after cold exposure with higher UCP1 expression in BAT (Fig. 3g, h, j). However, the browning of iWAT by CQA treatment was dispelled after antibiotics intervention (Fig. 3h, i). These results suggested that iWAT might be the primary target tissue for CQA to enhance thermogenesis and improve whole-body metabolism by modulating gut microbiota, whereas the upregulation of UCP1 in BAT is unlikely to be controlled by the microbial regulatory route.

To further clarify the causative role of the gut microbiota in mediating the beneficial effects of CQA on metabolic diseases, we transplanted fecal microbiota from vehicle- or CQA-treated mice into recipient mice for 8 weeks to ensure engraftment of the donor microbiome. We performed a 16S rRNA sequencing analysis and compared the gut microbiota between the donor groups (HFD-V and HFD-CQA), as well as the gut microbiota between recipient groups (rHFD-V and rHFD-CQA). In the PCA plot, the two donor groups were clearly separated by the first component (PCA1). After FMT, the two recipient groups were also separated by PCA1, and the distribution of the rHFD-V group was closer to the HFD-V group while the rHFD-CQA group was closer to the HFD-CQA group in the plot (Fig. S3i), suggesting the successful FMT in recipient mice. Compared with the control microbiota-transplanted (rHFD-V) mice, CQA microbiota transplantation (rHFD-CQA) did replicate the effects of CQA treatment on metabolic dysfunctions. Consistent with body weight control (Fig. 3k), the metabolic capacity of glucose and lipid metabolism exhibited a noticeable improvement after receiving CQA-treated microbiota (Fig. 3l–n, Fig. S3k-o). Besides, rHFD-CQA mice markedly reduced hepatic lipogenesis and lipid accumulation (Fig. 3o, p, Fig. S3p). Additionally, compared with rHFD-V mice, rHFD-CQA mice were more resistant to body temperature loss under cold stress via iWAT browning, without significantly influencing UCP1 expression in BAT (Fig. 3q, r). Meanwhile, the cold-challenged rHFD-CQA mice displayed a significant increase in the protein levels of UCP1 only in iWAT (Fig. 3s), which coincided with the finding from the antibiotics study that only the difference in UCP1 expression in iWAT was diminished by antibiotics-induced microbiota depletion. Hence, both microbiota depletion and FMT experiments supported the notion that the gut microbiota plays a role in the anti-obesity effects of CQA, and FMT from CQA-treated DIO-mice is sufficient to improve systematic metabolism by promoting iWAT browning to increase energy metabolism.

### CQA reversed obesity-induced gut dysbiosis and enriched lactic acid-producing bacteria

**Fig. 4:**
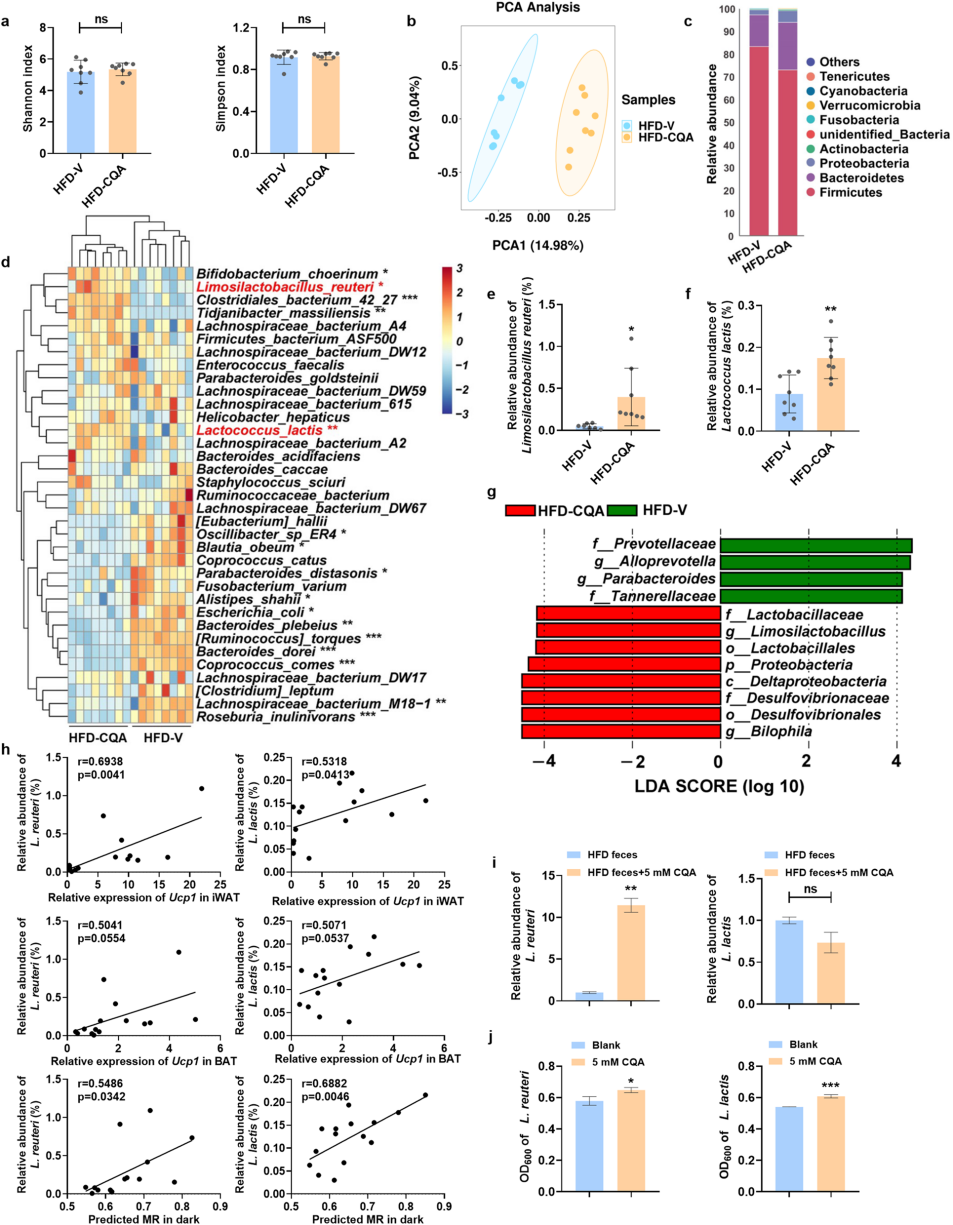
CQA alters gut microbiota compositions, especially in lactic acid bacteria enrichment. **a** Alpha diversity analyses based on Shannon index and Simpson index. **b** Principal component analysis. **c** Bacterial taxonomic profiling in phylum levels. **d** Heatmap of bacterial taxa. **e**, **f** Relative abundance of *L. reuteri* (%) (**e**) and *L. lactis* (%) (**f**) in feces detected by sequencing analysis. **g** Discriminative taxa determined by LEfSe between two groups (log10 LDA > 4). **h** Correlation of the abundance of *L. reuteri* (%) and *L. lactis* (%) with thermogenesis traits, including *Ucp1* mRNA expression in adipose tissues and after 4 h cold stress and predicted MR. **i** Relative abundance of *L. reuteri* and *L. lactis* in anaerobic cultivation of fecal microbiota from DIO mice. **j** OD_600_ of *L. reuteri* and *L. lactis*. **a**–**g** *n* = 7–8/group. **h** *n* = 15–16/group. **i**, **j** *n* = 3/group. Data are presented as mean ± SD. *p < 0.05; **p < 0.01; ***p < 0.001. ns means not statistically significant

We next investigated how CQA affected gut microbiota structure via 16S rRNA gene amplicon sequencing. CQA did not change α-diversity compared with DIO mice without treatment (Fig. 4a), whereas the overall structure of the gut microbiota between HFD-V and HFD-CQA was different (Fig. 4b). Phylum-level taxonomic profiling showed no significant differences after CQA treatment (Fig. 4c). Among the top 35 most abundant species with a relative abundance of greater than 0.1% in DIO mice, lactic acid-producing bacteria (LAB), especially *Limosilactobacillus reuteri* (*L. reuteri*) and *Lactococcus lactis* (*L. lactis*), were significantly enriched after CQA treatment (Fig. 4d–g). Correlations between the occurrence of these two LAB and *Ucp1* mRNA in adipose tissues or predicted MR showed that thermogenesis was also enhanced along with LAB enrichment (Fig. 4h). Similarly, we found that the abundance of *L. reuteri* was also enriched in rHFD-CQA group after FMT (Fig. S3j). To identify which LAB species specifically responded to CQA treatment, mouse microbiota and single bacterial strains were incubated in vitro with CQA. CQA supported the growth of *L. reuteri* in anaerobic cultivation of fecal microbiota from DIO mice, whereas *L. lactis* did not respond to CQA (Fig. 4i). CQA also stimulated the growth of both *L. reuteri* (ATCC 23,272) and *L. lactis* (ATCC 19,435) (Fig. 4j). Thus, our results indicated that CQA significantly alters cecal microbiota composition and that *L. reuteri* is substantially enriched. However, *L. reuteri* could not hydrolyze CQA to CA (Fig. S2c), suggesting that the enhanced growth of *L. reuteri* by CQA did not directly result from the metabolism of CQA by the microbe.

Next, we selected six mice from the long-term HFD-CQA group (Fig. 1) and designated the top three mice with the greatest body weight gain as non-responders and the top three mice with the least body weight gain as responders (Fig. S4a). Compared to non-responders, CQA treatment exhibited better improvements in body weight control in responders. Responders displayed lower fat/lean index levels and serum LDL-C levels than non-responders. However, other parameters such as fasting blood glucose, serum triglyceride, and serum T-CHO, tended to change without significant differences, which might be due to the insufficient sample numbers (Fig. S4b-f). Interestingly, the CQA-responding microbe, *L. reuteri* but not *L. lactis*, was highly enriched in the responders (Fig. S4g, h).

**Fig. 5:**
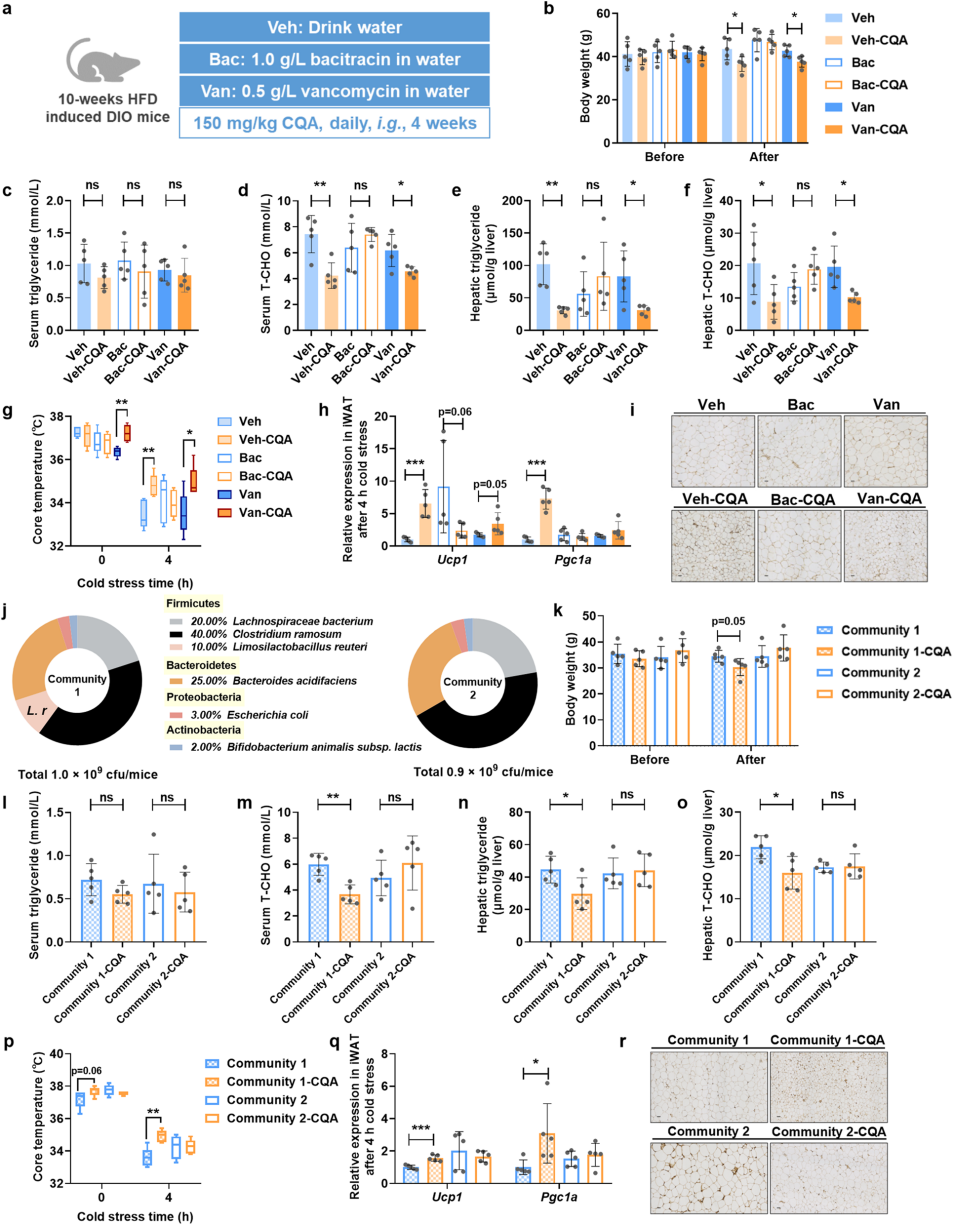
Intervention of mice with microbial communities lacking or including *L. reuteri* influence the anti-obesity phenotypes of CQA. The mice were fed a HFD for 10 weeks and then gavaged with vehicle or CQA (150 mg/kg) together with selective antibiotic in drinking water for 4 weeks. **a** Schematic diagram of the selective antibiotic supplementation in combination with CQA. **b** Body weight. **c** Serum triglyceride. **d** Serum T-CHO. **e** Hepatic triglyceride. **f** Hepatic T-CHO. **g** Core temperature. **h** Relative mRNA expression of thermogenic genes in iWAT. **i** Representative UCP1 staining of iWAT sections, scale bar: 50 μm. The mice were fed a HFD for 10 weeks and then gavaged with vehicle or CQA (150 mg/kg) together with defined microbiota colonization for 4 weeks. **j** Composition of the defined microbiota for the respective experimental groups. **k** Body weight. **l** Serum triglyceride. **m** Serum T-CHO. **n** Hepatic triglyceride. **o** Hepatic T-CHO. **p** Core temperature. **q** Relative mRNA expression of thermogenic genes in iWAT. **r** Representative UCP1 staining of iWAT sections, scale bar: 50 μm. *n* = 5/group. Data are presented as mean ± SD. *p < 0.05; **p < 0.01; and ***p < 0.001. ns means not statistically significant

To verify the role of *L. reuteri* in the regulatory effects of CQA, we manipulated the composition of gut microbiota in DIO mice and then administrated the mice with CQA. In in vitro cultures, we screened the susceptibility of *L. reuteri* to the component of antibiotics cocktail and other common antibiotics, and *L. reuteri* was more susceptible to each component in the cocktail, especially bacitracin, but was not affected by vancomycin (Fig. S5a-e). Therefore, bacitracin and vancomycin were used to selectively interfere with *L. reuteri* and thereby generate mice lacking (bacitracin-treated) or enriched in (vancomycin-treated) *L. reuteri* (Fig. 5a, Fig. S5f). The results showed that CQA still decelerated body weight gain and alleviated dyslipidemia and hepatic steatosis in vancomycin-treated (Van-CQA) mice, but not in bacitracin-treated (Bac-CQA) mice (Fig. 5b–f, Fig. S6a, b), indicating that the presence of *L. reuteri* might be required for the action of CQA in ameliorating obesity-associated disorders. Moreover, Veh-CQA mice and Van-CQA mice were more resistant to cold-induced body temperature loss along with the upregulation of UCP1 expression in iWAT (Fig. 5g–i), which was not observed in the Bac-CQA group. The results suggested that the lack of *L. reuteri* by bacitracin treatment might counteract the effect of CQA on thermogenesis.

Because both bacitracin and vancomycin strongly affected the overall structure of gut microbiota in addition to *L. reuteri*, we further evaluated the role of *L. reuteri* in regulating the effects of CQA by using simplified microbial communities consisting of six bacterial strains (Fig. 5j). We colonized antibiotics-treated DIO mice with customized microbial communities either containing *L. reuteri* (Community 1) or not containing *L. reuteri* (Community 2) for 1 week prior to CQA treatment. Compared with the Community 2-colonized mice, the positive effect of CQA treatment on metabolic dysfunctions was replicated in Community 1-colonized mice (Fig. 5k–o, Fig. S6c, d). The Community 1-colonized mice were also more sensitive to CQA administration in terms of body temperature maintained under cold stress by promoting beiging of iWAT (Fig. 5p–r). Hence, both selective microbiota depletion and customized microbial community colonization supported the notion that *L. reuteri* plays a role in the anti-obesity effects of CQA, especially in functions related to thermogenesis and energy metabolism.

### *L. reuteri* synergized with low-dose CQA in the treatment of obesity and associated metabolic dysfunctions

Human gut commensal LAB are Gram-positive bacteria that have been extensively studied [29]. The pharmacological importance of LAB as probiotics is evidenced by their ubiquitous contribution to the healthy microecology of animal and host immunity [30]. To verify that obesity-associated conditions could benefit from *L. reuteri* or *L. lactis* colonization, DIO mice were orally administered with *L. reuteri* or *L. lactis* or saline twice per week for 8 weeks (Fig. S7a). Unexpectedly, single-microbe treatment with *L. reuteri* failed to produce anti-obesity effects as observed in CQA treatment, whereas treatment with *L. lactis* significantly prevented obesity and associated traits (Fig. S7b-l). Taken together, these data identified *L. lactis* as a potential probiotic for the treatment of obesity-related dysfunctions, whereas *L. reuteri* had no direct effects.

**Fig. 6:**
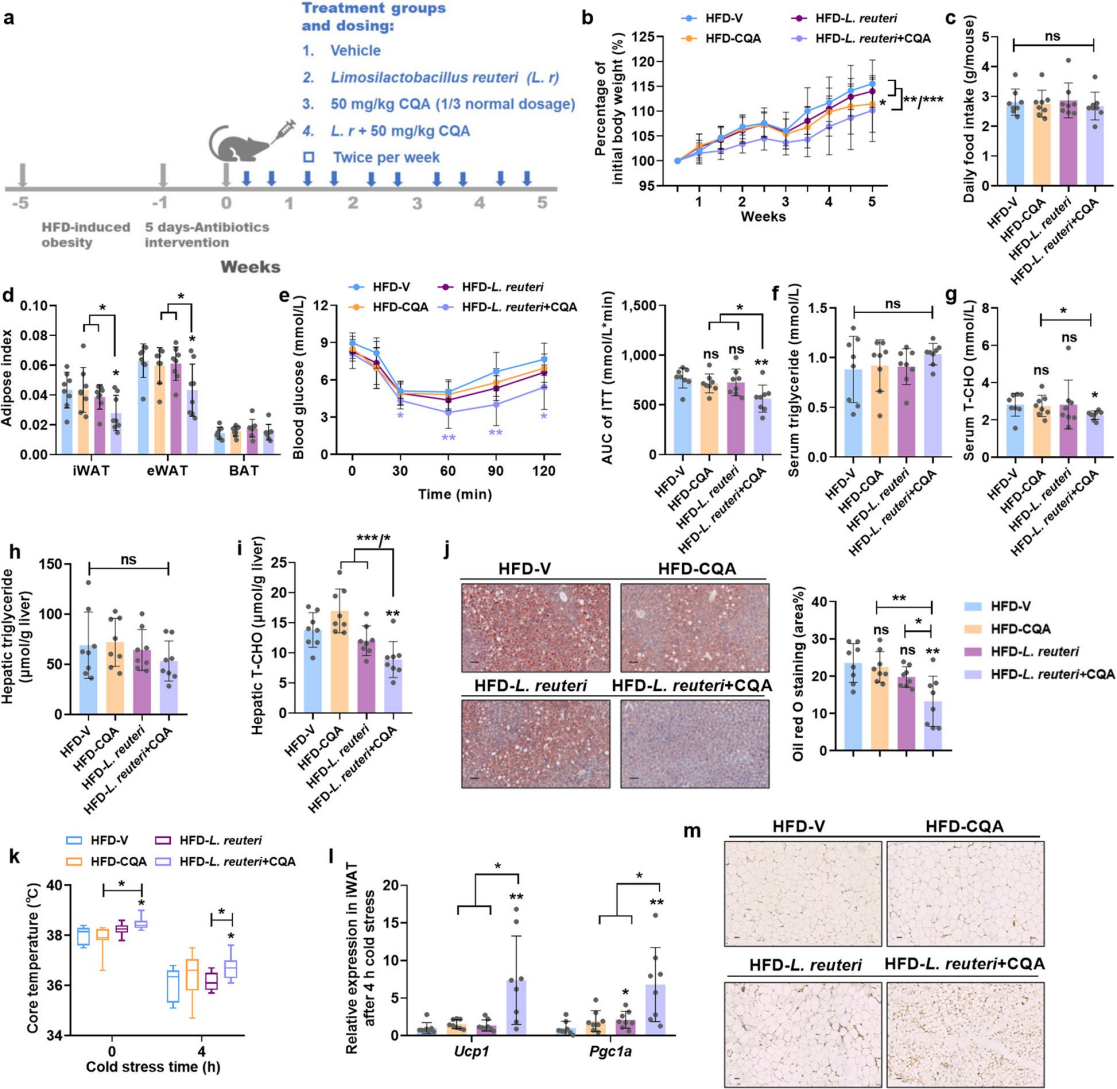
*L. reuteri* improves metabolic disorders in DIO mice treated with CQA. HFD-fed mice were treated twice per week with *L. reuteri* + CQA (1 × 10^8^ CFU bacteria, 50 mg/kg CQA) by oral gavage for 5 weeks. **a** Schematic diagram of the *L. reuteri* supplementation in combination with CQA. **b** Body weight change. **c** Daily food intake. **d** Adipose index. **e** ITT and AUC. **f** Serum triglyceride. **g** Serum T-CHO. **h** Hepatic triglyceride. **i** Hepatic T-CHO. **j** Representative Oil red O staining of liver sections, scale bar: 50 μm. Lipids were stained positive (red color) with Oil red O and quantified by Image J software. **k** Core temperature. **l** Relative mRNA expression of thermogenic genes in iWAT. **m** Representative UCP1 staining of iWAT sections, scale bar: 50 μm. *n* = 8/group. Data are presented as mean ± SD. *p < 0.05; **p < 0.01; ***p < 0.001. ns means not statistically significant

These results prompted us to determine the potential efficacy of combined *L. reuteri* and CQA treatment in DIO mice, as CQA might support the growth and/or metabolism of *L. reuteri*. To minimize the anti-obesity effects elicited by CQA, the dosage regimen was adjusted from daily dosing to twice per week dosing, with a CQA dose that decreased from 150 to 50 mg/kg (Fig. 6a). Neither single administration of low-dose CQA nor *L. reuteri* had significant therapeutic efficacy on metabolic parameters (Fig. 6b–m, Fig. S8). In contrast, the combined therapy markedly reduced body weight gain (Fig. 6b) without influencing food intake (Fig. 6c) when compared to the control (HFD-V) group and the single therapy groups. Accordingly, the adipose index (Fig. 6d), glucose metabolism (Fig. 6e, Fig. S8a) and serum and hepatic biochemical parameters associated with lipid metabolism (Fig. 6f–i, Fig. S8b-d) were significantly improved in *L. reuteri* + CQA-treated mice compared with either treatment alone. Moreover, the morphology of liver sections using Oil red O staining, and the results obtained from mRNA expression of hepatic lipid metabolism-related genes (Fig. 6j, Fig. S8e), together indicated that *L. reuteri* + CQA co-treatment attenuated liver steatosis. Notably, the co-treatment pronouncedly increased energy expenditure revealed by the higher core temperature after cold exposure (Fig. 6k). In addition, we found that the co-treatment promoted thermogenesis mainly through the browning of iWAT and BAT, as indicated by the increased mRNA levels of thermogenic-associated genes *Ucp1* and *Pgc1α*, as well as evident browning morphology (Fig. 6l, m, Fig. S8f-i). Collectively, these data demonstrated that the combination of *L. reuteri* and CQA exhibited a superior anti-obesity response than either treatment alone. Besides growth stimulation by CQA, we assumed that CQA might also affect the metabolism of *L. reuteri*, which then produces metabolites that contribute to the beneficial effects of CQA on obesity.

### CQA supplementation upregulated propionic acid production by *L. reuteri*

**Fig. 7:**
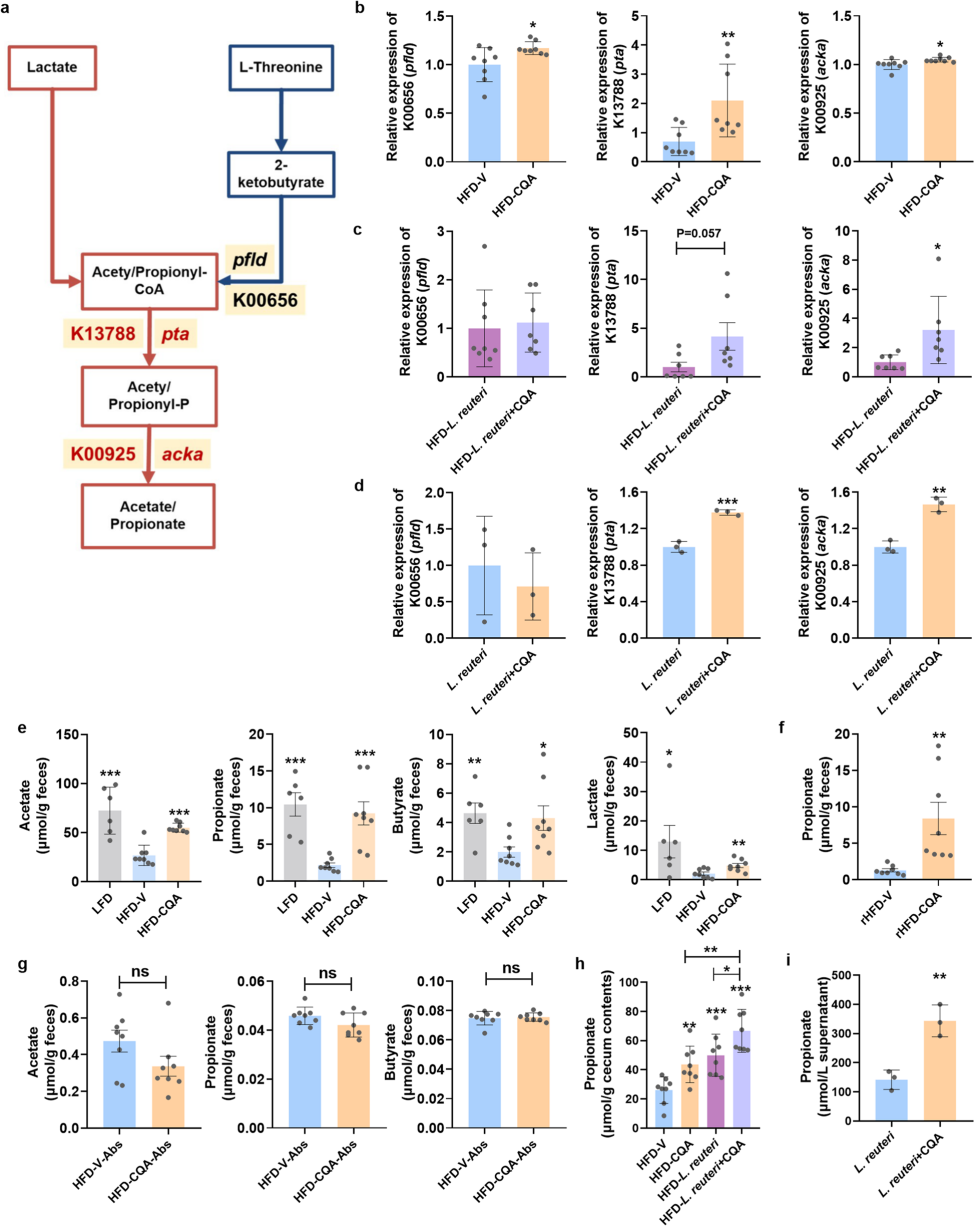
CQA administration increased fecal propionate level in DIO mice. **a** Metabolic pathway for acetate and propionate and corresponding KEGG Orthology (KO) and genes. **b** Fecal KEGG orthology (KO) expression of CQA-treated mice. **c** Fecal KO expression of *L. reuteri* + CQA-treated mice. **d** KO expression of CQA-treated *L. reuteri* in anaerobic cultivation. **e** Levels of acetate, propionate, butyrate, and lactate analyzed in feces after CQA-treatment. **f** Level of propionate analyzed in feces after FMT treatment. **g** Levels of acetate, propionate, and butyrate analyzed in feces in microbiota-reduced mice. **h** Level of propionate analyzed in cecum contents after *L. reuteri* + CQA co-treatment. **i** Level of propionate analyzed in supernatant of *L. reuteri* after co-cultured with CQA. **b**, **c**, **f**–**h** *n* = 7–8/group. **d**, **i** *n* = 3/group. **e** *n* = 6–8/group. Data are presented as mean ± SD. *p < 0.05; **p < 0.01; and ***p < 0.001. ns means not statistically significant

To investigate the metabolic and functional alterations of the gut microbiota induced by CQA, the Phylogenetic Investigation of Communities by Reconstruction of Unobserved States (PICRUSt) was employed to predict the functional orthology using Kyoto Encyclopedia of Genes and Genomes (KEGG) database. Compared with HFD-V group, the gut microbiome of CQA-treated mice was predicted to have a higher potential to produce acetate and propionate (Fig. 7a), which was further supported by the observation that relative abundance of K00656 (methylmalonyl-CoA mutase, *pfld*), K13788 (phosphate acetyltransferase, *pta*), and K00925 (acetate kinase, *acka*), the essential enzymes mediating both acetate and propionate synthesis, were increased by CQA treatment (Fig. 7b). Microbial expression of K13788 and K00925 were also upregulated either in the *L. reuteri* + CQA-treated mice (Fig. 7c) or in the *L. reuteri* + CQA co-cultures (Fig. 7d). These data implied that CQA supplementation might drive *L. reuteri* to generate more SCFAs, especially acetate and propionate.

SCFAs are the end products of microbial fermentation in the gut. Many reports have discovered the beneficial effects of SCFAs and lactate in attenuating metabolic diseases [31]. To support the above gene expression results and verify whether bacterial SCFAs and lactate were altered, a liquid chromatography/mass spectrometry (LC/MS)-based analysis was performed to measure these microbial metabolites in different models. As expected, acetate, propionate, butyrate, and lactate concentrations were all increased in the feces after long-term treatment with CQA, but abrogated in microbiota-reduced mice (Fig. 7e, g). However, these observations were not consistent with the KEGG Orthology (KO) changes, suggesting that the alteration of SCFAs and lactate might be the consequence of the attenuated gut dysbiosis given that other SCFAs-producing microbes were also recovered due to the improved metabolic environment. To further identify which metabolite was responsive to CQA treatment, we measured SCFAs and lactate in the model of FMT, short-term *L. reuteri* + CQA co-treatment, and anaerobic *L. reuteri* + CQA co-incubation. Notably, propionate was consistently and most highly elevated in all models (Fig. 7f, h, i). Besides, we also found that lactate production was increased in all models (Fig. S9a-c), which might be partially ascribed to *L. reuteri* enrichment by CQA treatment. Altogether, these findings provided a new perspective on the mutualistic interaction between CQA and *L. reuteri*, suggesting that CQA might enhance the bacterial potential of propionate production.

### Monocarboxylate transporter 1 was required for the propionate-induced energy expenditure in obese mice

**Fig. 8:**
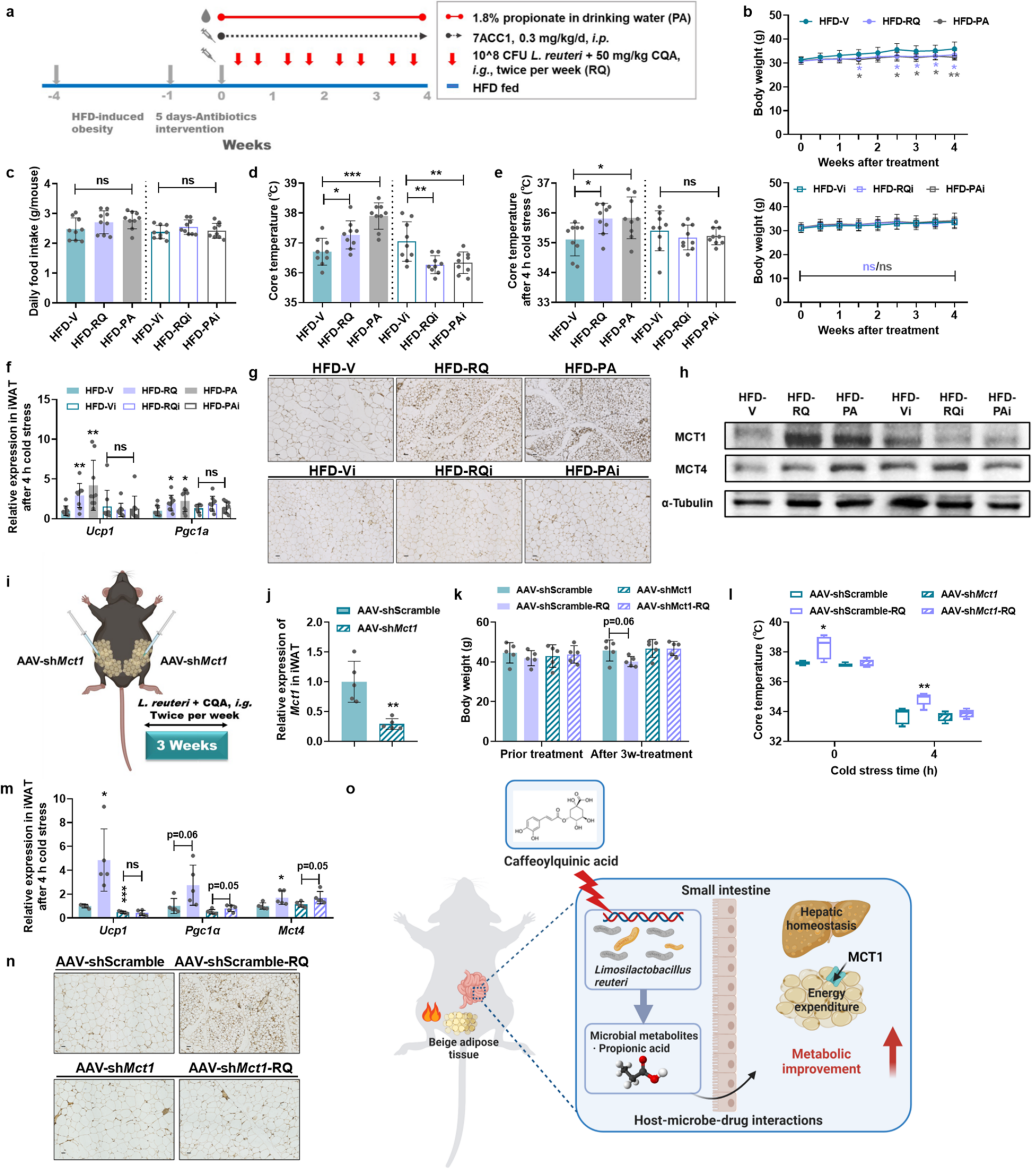
Monocarboxylate transporter 1 (MCT1) is the essential transport protein involved in propionate-induced energy expenditure. HFD-fed mice were treated with propionate and/or MCT inhibitor intervention for 4 weeks. **a** Schematic diagram of propionate supplementation and MCT inhibitor intervention. **b** Body weight. **c** Daily food intake. **d**, **e** Core temperature against room temperature (**d**) and cold stress (**e**). **f** Relative mRNA expression of thermogenic genes in iWAT. **g** Representative UCP1 staining of iWAT sections, scale bar: 50 μm. **h** Protein expression of MCT1 and MCT4 in iWAT. The mice were fed a HFD for 10 weeks and then treated with *L. reuteri* + CQA twice per week for 3 weeks after AAV injection. **i** Experimental design and iWAT AAV injection. **j** Relative mRNA level of *Mct1* in iWAT injected with AAV-sh*Mct1* or AAV-Scramble. **k** Body weight. **l** Core temperature. **m** Relative mRNA expression of thermogenic genes in iWAT. **n** Representative UCP1 staining of iWAT sections, scale bar: 50 μm. **o** Proposed model for the anti-obesity effects of caffeoylquinic acid (CQA) via *L. reuteri*-propionate-beige fat axis. **b**–**h** *n* = 9/group. **j**–**n** *n* = 5/group. Data are presented as mean ± SD. *p < 0.05; **p < 0.01; and ***p < 0.001. ns means not statistically significant. “RQ” means *L. reuteri* + CQA co-treatment; “PA” means propionate treatment; “i” means treating combined with MCT inhibitor 7ACC1

The data above clearly showed that CQA could stimulate the growth of *L. reuteri* and promote propionate production. To further explore the causative relationship between the increased propionate and the browning of iWAT, we administered sodium propionate in drinking water to DIO mice for 4 weeks (Fig. 8a). The results showed that the propionate supplementation (HFD-PA) produced effects similar to those of the combined use of *L. reuteri* and CQA (HFD-RQ) in DIO mice, but did not impact appetite (Fig. 8b, c, Fig. S10). Most importantly, propionate induced energy expenditure by promoting the thermogenic function of iWAT, which phenocopied the effects of *L. reuteri* + CQA co-treatment (Fig. 8d–g). The replenishment results further demonstrated that propionate might be a microbial metabolic signal that is selectively evoked by CQA treatment and contributes to the superior anti-obesity effects of *L. reuteri* + CQA co-therapy.

MCTs have been reported to drive SCFAs transport across the plasma membrane of adipocytes [32]. Among the four isoforms, MCT1 and to a lesser extent MCT4 are responsible for propionate transport [33]. Given recent evidence that the expression of MCT1/4 is controlled by physiological stimuli of energy expenditure [34], we found that MCT1 protein levels were indeed strongly upregulated in iWAT by propionate supplementation or *L. reuteri* + CQA co-treatment, whereas MCT4 expression was only slightly upregulated (Fig. 8h), suggesting that a potential role of MCT1 in propionate-induced thermogenesis. Accordingly, we investigated whether the blockage of MCT1 function could abolish the therapeutic effect of propionate or *L. reuteri* + CQA co-therapy on obesity. The MCT inhibitor (7ACC1) was administered to disrupt the propionate distribution in obese mice. The results revealed that 7ACC1-treated mice did not respond to either *L. reuteri* + CQA (HFD-RQi) or propionate treatment (HFD-PAi) in obesity-associated metabolic disorders (Fig. 8b, c, Fig. S10). In parallel, along with the absence of cold resistance and browning of iWAT by 7ACC1 intervention, the increasing trend of MCT1 expression was also diminished (Fig. 8d–h).

To obtain more definitive evidence for the crucial role of MCT1 in propionate-induced iWAT browning, we knocked down MCT1 in the iWAT through in situ injections of AAV-sh*Mct1* and then treated the mice with *L. reuteri* + CQA (Fig. 8i). Significantly downregulated *Mct1* mRNA was verified in the iWAT of AAV-sh*Mct1*-injected mice (Fig. 8j). Similar to 7ACC1 treatment, MCT1 knockdown abolished the efficacy of *L. reuteri* + CQA co-treatment, including body weight loss and thermogenic enhancement (Fig. 8k–n). The above results implied that *L. reuteri* synergizes with low-dose CQA to generate more propionate and thereby alleviates obesity-associated metabolic disorders in an MCT1-dependent manner (Fig. 8o).

## Discussion

Green coffee bean extracts are widely used as a dietary supplement for body weight control. Clinical studies in overweight subjects or patients with metabolic syndrome have shown that the consumption of green coffee bean extracts or a complex of CQA isomers has an ameliorative effect on insulin resistance and abdominal obesity [35, 36]. However, the underlying molecular mechanism remains still unclear. Herein, we applied CQA at the dose of 150 mg/kg for chronic treatment and 50 mg/kg for co-therapy with the microbe, which matches the dose in humans based on human-animal dose conversion [37, 38] and is therefore clinically relevant. Due to the poor absorption in the intestinal tract, CQA is usually in direct contact with gut microbiota after its oral administration. In the current study, we demonstrated that gut microbiota and its metabolites, such as propionate, induced by CQA played an essential role in the attenuation of obesity-related metabolic dysfunctions.

Energy homeostasis depends on brown or beige adipose tissues, which are considered a new therapeutic target for obesity treatment. As a vital element in thermogenesis, UCP1 converts energy generated by short-circuiting the mitochondrial electron transport chain to increase thermogenesis [39]. The brown and beige adipocytes in BAT and iWAT are known as the UCP1-positive adipocytes, which can initiate a thermogenic response to an acute cold challenge [40]. Generally, UCP1 is inherently inactive and requires concomitant activation to produce heat [41]. Our data suggested that CQA could stimulate UCP1 upregulation and activation in both iWAT and BAT to produce metabolic benefits. Furthermore, the results from microbiota depletion and FMT experiments supported that the upregulation of UCP1 in BAT might not be associated with microbial regulatory routes, whereas iWAT is the primary target tissue for CQA to enhance energy expenditure and improve whole-body metabolism by modulating gut microbiota. We also found that CQA upregulated *L. reuteri* abundance in both WT mice and UCP1 KO mice (data not shown), suggesting that UCP1 is the critical downstream effector in adipose tissue responding to the enrichment of *L. reuteri*. However, the underlying molecular mechanism regarding UCP1 activation needs further investigation.

Although the exact mechanism linking the gut microbiota to obesity is far less understood, many animal and human studies have implicated the distortion of gut microbial balance in obesity and related metabolic disorders [42]. The gut microbiota is a potential target in the management of obesity. Therefore, natural products could act as prebiotics to remodel gut microbiota, and thereby produce favorable metabolic outcomes. However, poor concordance in response to natural products is noted, which are often attributed to the large individual differences in the gut microbiota. In our study, we verified that a possible responder/non-responder effect did exist for long-term CQA treatment. However, the results from *L. reuteri* + CQA co-treatment study suggested that colonization of *L. reuteri* sensitized mice to CQA treatment, and therefore, the responder/non-responder effect was much weakened in the co-treatment group.

Several studies have revealed that short-term treatment with probiotics such as LAB could elicit anti-obesity effects in obese rodents, but long-term colonization failed due to tolerance issues [11, 43]. At the beginning of the current study, we took a great interest in two LAB, *L. reuteri* and *L. lactis*, since they are well recognized for their prebiotic functions and are enriched in the gut of CQA-treated DIO mice. However, only *L. lactis* attenuated metabolic dysfunction, whereas *L. reuteri* had negligible effects. These observations prompted us to further investigate the interplay between CQA and *L. reuteri*. Both selective microbiota depletion and customized microbial community colonization demonstrate that *L. reuteri* plays a crucial role in the anti-obesity effects of CQA. Moreover, supplementing *L. reuteri* with low-dose CQA as prebiotics showed add-on anti-obesity benefits compared to each treatment, thus highlighting the synchronous application of probiotics with a prebiotic such as CQA as a promising gut microbiota-targeting strategy for metabolic intervention. It should be noted that we did not include an inactivated-bacteria group as control, which is a limitation of our study.

Emerging evidence has demonstrated that SCFAs, the end fermentation products of endogenous lactate or pyruvate in anaerobic microbes, serve as powerful signal transmitters for the communication between the gut microbiota and host metabolism [44]. In the present study, the KOs in feces altered by CQA treatment were functionally related to acetate and propionate production. Nevertheless, it is not accessible to pinpoint which gene is responsible for each SCFA, especially as CoA-transferases tend to have broad substrate specificity [45]. In accordance, we only found that propionate production was consistently and most significantly altered in all experimental settings, suggesting that propionate might represent the key bacterial metabolite in the “CQA-microbiota-host metabolism” communication in our study. Two monocarboxylic acid transporters, MCT1 and MCT4, widely distributed in multiple organs, are reported to transport propionate across the adipocytes [46]. Indeed, upregulated MCT1 protein levels were observed in iWAT after *L. reuteri* + CQA co-treatment. Therefore, we conducted the MCT inhibitor 7ACC1 treatment or iWAT *Mct1* knockdown studies to determine whether the anti-obesity capability of *L. reuteri* + CQA co-therapy required propionate signaling in adipose tissues. Both interventions abolished the thermogenic enhancement induced by *L. reuteri* + CQA co-treatment. Hence, our current results implied that *L. reuteri*-derived propionate might function through an MCT1-dependent pathway and thus emphasized the functional importance of the *L. reuteri*-propionate-beige fat axis in the anti-obesity effect of CQA.

Propionate possesses multiple beneficial effects on host metabolic disorders, such as decreasing glycaemia, reducing appetite and abdominal fat accumulation, stimulating intestinal gluconeogenesis and thus improving glucose metabolism, attenuating systemic inflammation, and reverting HFD-induced effects on functional connectivity between the visual and auditory cortex [47–50]. However, despite the apparent body weight control, we did not observe any changes in appetite. These inconsistent results might be associated with the different dosages and the route of administration. Even though we supplied a relatively lower dose of propionate, it could still induce evident beiging morphology in iWAT and increase the energy expenditure, emphasizing the direct thermogenic potential of propionate to reduce obesity.

There are some limitations to our study. First, we recognized that CQA could significantly upregulate BAT’s thermogenic capacity, but it does not seem to be associated with microbial regulatory routes. It should be noted that our research does not exclude other mechanisms involved in the anti-obesity action of CQA [19–21]. Second, our study suggested a novel therapeutic strategy by the combinatory use of probiotic *L. reuteri* and prebiotic CQA in the treatment of obesity; however, knowledge of the molecular mechanism of how CQA promotes the growth of *L. reuteri* and selectively upregulates its propionate production is lacking. Third, propionate production is a function of a wide variety of gut commensals that are much more dominant than *L reuteri* in the gut of humans and mice, and we could not exclude other bacteria that play a role in the beneficial effects of CQA. Nevertheless, we demonstrated that the co-treatment of *L. reuteri* and low-dose CQA did reproduce the most beneficial effects of CQA, deciphering that CQA at least partially works through the *L. reuteri-*propionate-beige fat axis to treat obesity. More in-depth studies and microbial investigations are needed to verify our findings.

## Conclusions

In conclusion, the present work demonstrated that supplementation with CQA reverses HFD-induced obesity by modulating the composition and inducing “ubiquitous” metabolites, such as propionate which is at least partly responsible for the positive effects observed. The combination of *L. reuteri* and low-dose CQA shows superior beneficial effects on obesity than either treatment alone, which helps lower the dosage and dose frequency of CQA and strengthens the translational significance of our study. Overall, our findings delineate the deciding role of the *L. reuteri*-propionate-beige fat axis in the anti-obesity property of CQA and also provide a rationale for applying the microbial-based “probiotic-prebiotic” therapeutic strategy for the treatment of obesity-associated metabolic disorders. Our study here also opens a new window for understanding the in vivo action of herbal extracts from a microbiome-target perspective.

## Methods

### Chemicals and reagents

Caffeoylquinic acid, bacitracin, neomycin, streptomycin, and vancomycin were purchased from Dalian Meilun Biotech Co. Ltd (Dalian, China). Sodium propionate was purchased from Aladdin Reagent (Shanghai, China). MCT inhibitor 7ACC1 was purchased from MedChem Express (New Jersey, USA). SCFA standards for LC–MS analysis including acetic, propionic, butyric, and lactic acids were obtained from Macklin Reagent (Shanghai, China). O-Benzylhydroxylamine hydroxylamine hydrochloride (O-BHA), N-(3-dimethylaminopropyl)-N’-ethylcarbodiimide hydrochloride (EDC), and d3-AA were purchased from Sigma-Aldrich (Missouri, USA). CER (17:0) was purchased from Avanti Polar Lipids (Alabaster, USA). UCP1 antibody (GB112174) was purchased from Servicebio (Wuhan, China). MCT1 antibody (20,139–1-AP) and MCT4 antibody (22,787–1-AP) were purchased from Proteintech (Wuhan, China). α-Tubulin antibody (SC-5286) was purchased from Santa Cruz (CA, USA). HRP-conjugated anti-mouse IgG (7076S) and anti-rabbit IgG (7074S) were purchased from Cell Signaling Technology (MA, USA). A high-fat diet (HFD, 60 kcal%, D12492) and a matched low-fat diet (LFD, 10 kcal%, D12450J) were purchased from Research Diets Inc. (New Jersey, USA).

### Animal studies

Six- to eight-week-old C57BL/6 J male mice were purchased from HuaFukang BioScience Company (Beijing, China). UCP1 knockout (UCP1 KO) mice and the control wild-type (WT) mice were kindly provided by Professor Jiqiu Wang at Shanghai Jiao Tong University School of Medicine. All the mice were housed in a pathogen-free facility under a 12-h dark–light cycle with ad libitum access to food and water.

For chronic treatment, sample size estimation was performed before experiments. Adult male C57BL/6 J mice were exposed to a HFD or LFD diet for 8 weeks. After 8 weeks of feeding, mice were randomized and assigned to the following treatment groups: HFD-V, HFD-CQA (150 mg/kg/day), and LFD, and then subjected to daily intragastric treatment for an additional 8 weeks. UCP1 KO mice and control wild-type mice were exposed to a HFD diet for 4 weeks, and then subjected to daily intragastric treatment for an additional 4 weeks. CQA was dissolved in saline, and the pH was adjusted to 5–6 using sodium hydroxide. During the treatment, the body weight and food/water intake were monitored.

For gut microbiota depletion treatment, mice were administered the antibiotics cocktail (bacitracin, neomycin, and streptomycin) in drinking water at 0.1% (w/v) of each compound along with saline or 150 mg/kg CQA daily.

For fecal transplantation, donor mice were treated as described for chronic treatment. After 2 weeks of treatment, feces were collected twice per week for the subsequent 8 weeks under a laminar flow hood under sterile conditions. Feces from donor mice of each group were pooled and resuspended in sterile ice-cold saline. The solution was vigorously vortexed for 30 s, before natural sedimentation for 1 min. The supernatant was collected within 10 min before oral gavage to prevent changes in bacterial composition. Recipient mice were fed a HFD for 7 weeks, followed by 5-day antibiotics cocktail treatment, and then transplanted twice per week with fresh fecal slurry by oral gavage for 8 weeks.

For selective antibiotics treatment, DIO mice were fed with HFD for 10 weeks, followed by giving the antibiotic (0.1% (w/v) bacitracin, or 0.05% (w/v) vancomycin) in drinking water, respectively, along with daily administration of saline or 150 mg/kg CQA for 4 weeks.

For simplified microbial community colonization, DIO mice were colonized with a simplified bacterial community (Community 1) consisting of *Lachnospiraceae bacterium*, *Clostridium ramosum*, *Limosilactobacillus reuteri*, *Bacteroides acidifaciens*, *Escherichia coli*, and *Bifidobacterium animalis subsp. lactis*. A second group of DIO mice was colonized with Community 2, which consists similar bacterial members except for *L. reuteri*. Bacterial strains of the microbial community were selected based on the following criteria: reported to be altered in obesity patients or to affect experimental obesity, showed high abundance in 16S rRNA amplicon sequencing data of DIO mice, and were able to form a stable community in rodents. DIO mice were fed with HFD for 9 weeks, followed by 5-day antibiotics cocktail treatment, before being colonized with 1.0 × 10^9^ CFU/mice by oral gavage twice per week for 4 weeks. The composition of the bacterial consortia differed between Community 1 and 2 as shown in Fig. 5j. Along with colonization, the mice were daily administrated of saline or 150 mg/kg CQA for 4 weeks.

For single-microbe treatment, DIO mice were fed with HFD for 7 weeks, followed by 5-day antibiotics cocktail treatment prior to assignment to the following four treatment groups: HFD-V (gavaged with saline), HFD-*L. reuteri* (10^8^ CFU/mouse, suspended in saline), and HFD-*L. lactis* (10^8^ CFU/mouse, suspended in saline). Each group was treated twice per week for 8 weeks.

For co-administration, DIO mice were fed with HFD for 4 weeks, followed by 5-days antibiotics cocktail-treatment prior to assignment to the following four treatment groups: HFD-V (gavaged with saline), HFD-CQA (50 mg/kg), HFD-*L. reuteri* (10^8^ CFU/mouse), and HFD-*L. reuteri* + CQA. Each group was treated twice per week for 5 weeks.

For metabolite replenishment and inhibitor treatment, DIO mice were fed with HFD for 3 weeks, followed by 5-day antibiotics cocktail treatment prior to assignment to the following six treatment groups: HFD-V (gavaged with saline), HFD-RQ (10^8^ CFU of *L. reuteri* plus 50 mg/kg CQA), HFD-PA (gavaged with 1.8% sodium propionate in drinking water, w/v) with or without 7ACC1 (0.3 mg/kg/d, *i.p.*) treatment for 4 weeks.

For adeno-associated virus (AAV) injections, DIO mice were fed with HFD for 10 weeks, followed by 2.0 × 10^11^ vg virus-packed mouse Mct1 (m*Mct1*)-shRNA (AAV-sh*Mct1*) or control (AAV-shScramble) (Vector OBiO, Shanghai, China) injection at multiple sites on the inguinal fat pads. Three weeks after injection, antibiotics cocktail-treated mice were given *L. reuteri* (10^8^ CFU/mouse) plus CQA (50 mg/kg) or saline twice per week for 3 weeks.

### Glucose tolerance test

Glucose tolerance tests were performed after 16-h fasting. Blood samples were taken from the tail tip at 0, 15, 30, 60, 90, and 120 min after oral gavage of glucose (2 g/kg body weight), and blood glucose concentrations were measured with blood sugar test paper and glucometer (Sannuo Biosensors, China).

### Insulin tolerance test

For the insulin tolerance test, insulin (0.75 U/kg body weight) was administered via an intraperitoneal injection after 4-h fasting. All of the ITT tests were performed at indicated times the same as GTT.

### Body composition and indirect calorimetry

Body fat and lean mass were determined by ^1^H-NMR spectroscopy (Minispec LF90 II, Bruker, Karlsruhe, Germany) following the manufacturer’s protocol. Indirect calorimetry was performed on mice after 8-week CQA treatment using a 16-chamber indirect calorimeter (TSE PhenoMaster, TSE system, Germany) with one mouse per chamber as previously described [51]. Oxygen consumption (V_O2_) and carbon dioxide production (V_CO2_) were recorded, and heat production and RER (V_CO2_/V_O2_ ratio) were calculated. Mass-adjusted metabolic rate (MR) ratios were tested by global regression-based analysis-of-covariance (ANCOVA) using body mass as a covariate.

### Core temperature measurement

Mice were exposed to acute cold stress (4 ± 1 °C) in the artificial climate chamber (Yanghui, Ningbo, China) for 4 h, and their rectal temperature was measured at the indicated time points with a temperature feedback system (RWD; Shenzhen, Guangdong, China).

### Fasting blood glucose and fasting insulin assays

Fasting blood glucose was measured after 8-h fasting. Serum fasting insulin concentration was assessed in a 96-well microplate using an insulin mouse ELISA kit purchased from ThermoFisher (PA, USA). Homeostatic model assessment-insulin resistance (HOMA-IR) index was calculated as follows:

$$\text{HOMA-IR} = {\text{FBG}} \times \text{FINS/22.5}$$

$$\text{(FBG, mmol/L and FINS, mIU/L)}$$

### Biochemical analyses

For serum indices, levels of triglyceride, T-CHO, HDL-C, and LDL-C were measured according to the instruction of each assay kit (Jiancheng, Nanjing, China). For analysis of liver lipid content, 20 mg of the frozen liver was homogenized using a hybrid grinding machine (Biheng Bio-Technique Co. Ltd., Shanghai, China) in 10% (w/v) 50 mmol/L Tris with 1% Triton X-100. After centrifugation, supernatants were quantified using each assay kit (Jiancheng, Nanjing, China).

### RNA extraction and quantification

Total RNA was isolated from tissues with TRIzol reagent (Invitrogen, California, USA). cDNA was synthesized with PrimeScript Reverse Transcriptase (Takara Bio, Japan) according to the manufacturer’s instructions. Real-time PCR was performed using SYBR Premix Ex Taq (Takara Bio, Japan) on a CFX384 Touch Real-Time PCR Detection System (Bio-Rad, CA, USA). The results were analyzed by the ΔCt method and normalized to reference genes. The sequence of primers is shown in Supplemental table 1.

### Protein extraction and western blotting

Total protein was extracted using RIPA lysis buffer containing phenylmethylsulfonyl fluoride (PMSF) and phosphorylase inhibitor. Protein concentration was determined using the BCA Protein Assay Kit. Protein from the indicated samples was separated by sodium dodecyl sulfate-polyacrylamide gel electrophoresis (SDS-PAGE) and transferred onto polyvinylidene difluoride (PVDF) membrane. The blots were incubated with the respective primary antibody. After washing, anti-rabbit IgG was used as the secondary antibody. The blots were rewashed and developed by chemiluminescence (Immobilon Western Chemiluminescent HRP substrate, Millipore, MA, USA).

### Histology and microscopy

H&E staining or Oil red O staining was performed on formalin-fixed paraffin-embedded or OCT-embedded sections using a standard protocol, respectively. Brightfield images were scanned with the NanoZoomer 2.0-HT slide scanner (Hamamatsu, Japan) at different magnifications.

Immunohistochemical staining was performed on formalin-fixed paraffin-embedded sections using a standard protocol. Briefly, slides were dewaxed with xylene 3 times for 15 min each, hydrated through an alcohol gradient, and soaked in DPBS for 5 min. Antigen was recovered by pre-heating in the EDTA buffer (pH 8.0). Endogenous peroxidase activity was blocked with 3% H_2_O_2_ in PBS for 25 min. Non-specific binding sites were blocked using 3% bovine serum albumin in PBS. The slides were incubated overnight at 4 °C with rabbit polyclonal anti-UCP1 primary antibody diluted at 1:200 in PBS. According to the manufacturer’s instructions, the slides were rinsed in distilled water, followed by treatment with a secondary antibody (HRP labeled). Immuno-visualization was performed with 3,3’-diaminobenzidine (DAB) as substrate and counterstained with hematoxylin. Slide digital images were scanned with the NanoZoomer 2.0-HT slide scanner (Hamamatsu, Japan) at different magnifications.

### DNA isolation and 16S rRNA amplicon sequencing

Cecum contents or fecal samples of mice were collected and immediately frozen at − 80 °C. Metagenomic DNA was extracted using a fecal DNA isolation kit (QIAGEN, Dusseldorf, Germany). The concentration and quality of the DNA were assessed using a NanoDrop instrument and agarose gel electrophoresis, respectively. Sterile water was served as the negative control.

The universal primers 341F (5′-CCTAYGGGRBGCASCAG-3′) and 806R (5′-GGACTACNNGGGTATCTAAT-3′) were used to amplify the V3-V4 regions of the 16S rRNA gene. All PCRs were performed with 15 μL of Phusion® High-Fidelity PCR Master Mix (New England Biolabs, USA), 0.2 μmol/L of forwards and reverse primers, and approximately 10 ng of template DNA. Thermal cycling consisted of initial denaturation at 98 °C for 1 min, followed by 30 cycles of denaturation at 98 °C for 10 s, annealing at 50 °C for 30 s, and elongation at 72 °C for 30 s. Finally, the samples were incubated at 72 °C for 5 min. Sequencing libraries were generated using the TruSeq® DNA PCR-Free Sample Preparation Kit (Illumina, USA) following the manufacturer’s recommendations, and sequenced on the NovaSeq PE250 platform (Illumina, USA).

16S rRNA gene sequencing analysis was performed using QIIME (Quantitative Insights Into Microbial Ecology, http://qiime.org/). Briefly, sequence analysis was performed by Uparse software (v7.0.1001) for operational taxonomic units (OTUs) production. Each representative sequence was then assigned taxonomy against SILVA 132 database with 97% identity. The Mothur method and the SSUrRNA database were used to assign taxonomic categories to all OTUs at a confidence threshold of 0.8. Analyses for alpha diversity indices were calculated with QIIME and displayed with R programming language (v2.15.3). Cluster analysis was performed by principal component analysis (PCA) using the stats package (v3.5.0) and ggplot2 package (v3.2.0) in R programming language. Bacterial taxonomic profiling is displayed in a bar plot at the phylum level and in a heatmap at the species level using stats package (v3.6.3) and pheatmap package (v1.0.12). A linear discriminant analysis (LDA) column chart was generated to detect differentially abundant taxa across groups using the OmicStudio tools (https://www.omicstudio.cn/tool/).

### Bacterial strains and culture conditions

*Limosilactobacillus reuteri* subsp. *reuteri* (ATCC 23272) and *Lactococcus lactis* subsp. *lactis* (ATCC 19435) were grown at 37 °C under anaerobic conditions (anaerobic gas mixture, 80% N_2_, 10% CO_2_, and 10% H_2_) in MRS (Hope Bio-Technology Co., Ltd., China) broth. *Lachnospiraceae bacterium* (BNCC 354474), *Clostridium ramosum* (ATCC 25582), and *Bacteroides acidifaciens* (BNCC 353574) were grown at 37 °C under anaerobic conditions in trypticase soy broth with defibrinated sheep blood (ATCC® Medium #260). *Escherichia coli* (ATCC 25922) was aerobically grown at 37 °C in Luria-Bertani medium. *Bifidobacterium animalis subsp. lactis* (JCM 10602) was grown at 37 °C under anaerobic conditions in BBL medium (Hope Bio-Technology Co., Ltd., China). Microbial growth was monitored by measuring the optical density (OD_600_) and the growth curve was plotted. Common antibiotics, including neomycin, streptomycin, bacitracin, and vancomycin, were added separately for screening the susceptibility of *L. reuteri*. The feces from DIO mice were homogenized and cultured at 37 °C under anaerobic conditions in GAM (Gifu Anaerobic Medium, Hope Bio-Technology Co., Ltd. China) broth.

### Lipidomics

Serum lipidomics was performed based on an established protocol [52]. Briefly, a 50 μL aliquot of serum was extracted with 200 μL of ice-cold chloroform: methanol (2:1, v/v) solution containing CER (17:0) as an internal standard. The samples were vibrated and incubated for 20 min at 37 °C. The organic and aqueous phases were separated by centrifugation at 14,000 rpm for 15 min. The lower organic phase was transferred to another clean tube and evaporated to dryness at room temperature under vacuum. The residue was dissolved in 50 μL of chloroform: methanol (1:1, v/v) and then diluted with isopropanol: acetonitrile: H_2_O (2:1:1, v/v/v) before LC/MS analysis. Lipidomics analysis was performed on a Waters UPLC-ESI-Q/TOF MS (Waters Corp., MA) equipped with an electrospray ionization source. Separation was achieved on an Acquity UPLC CSH C18 column (100 × 2.1 mm, 1.7 μm, Waters Corp.). The mobile phase was a water/acetonitrile solution containing 10 mmol/L ammonium formate (2:3, A phase) and acetonitrile/isopropanol containing 10 mmol/L ammonium formate (1:9, B phase). The column temperature was maintained at 55 °C and the flow rate was 0.4 mL/min. Mass spectrometry data were acquired in negative ESI mode at a range of m/z 100–1500.

### Short-chain fatty acids analysis

The method of SCFAs determination was modified based on an established protocol [53]. Briefly, for feces or cecum contents, 10 mg samples were homogenized in 0.2 mL of extraction solution (200 μmol/L d3-AA as an internal standard in 50% aqueous methanol, 1:20, w:v) for 5 min, followed by centrifugation at 14,000 rpm, 4 °C for 10 min. An aliquot of 80 μL of supernatant was incubated with 10 μL of 0.1 mol/L O-BHA in methanol and 10 μL of 0.25 mol/L EDC in methanol at 25 °C for 1 h. After the incubation, the slurry exact was diluted by twofold in 50% aqueous methanol. An aliquot of 200 μL of diluted sample was extracted by 600 μL of chloroform with 10 min of vigorous shaking. After the centrifugation, 100 μL of the lower organic layer was transferred and evaporated to the dryness by a MiniVac system (Gene Company Limited, USA). The residue was reconstituted in 100 μL of 50% aqueous methanol, briefly vortexed, and centrifuged. Then, 5 μL of the sample was injected for LC–MS/MS. For microbial supernatant extraction, 20 μL of supernatant was precipitated by adding 60 μL of extraction solution. The resulting mixture was shaken briefly and centrifuged at 14, 000 rpm for 10 min at 4 °C. Then, 60 μL of supernatant was mixed with 20 μL of water prior to the derivatization and reconstitution described above.

A Thermo Scientific™ Vanquish™ Flex UHPLC system (Thermo Fisher Scientific, Germering, Germany) was coupled with an Orbitrap Elite™ mass spectrometer (LC–MS/MS). Chromatographic separation was performed on an Acquity UPLC® BEH C18, 50 × 2.1 mm, with a particle size of 1.7 μm (Waters, USA). Here, 0.1% formic acid in water with 10 mmol/L ammonium formate (phase A) and 0.1% FA in methanol: isopropanol (9:1 v/v, phase B) served as mobile phases with a flow rate of 0.4 mL/min. The separation was achieved by gradient elution. Mass spectrometry data were acquired in positive ESI modes at a range of m/z 100–1000.

### Statistical analysis

The data obtained are presented as the mean ± SD and displayed using GraphPad Prism 8.0 program (GraphPad Software, San Diego, Canada). When comparing two groups, statistical significance was determined using a two-tailed Student’s *t*-test. When more than two groups were investigated, a one-way analysis of variance with Tukey’s correction was applied for comparisons between different groups. The QIIME platform (v1.9.1) and R language packages were used for microbiota analysis. *P* values < 0.05 were considered statistically significant.

## Supplementary Information

**Additional file 1: Figure S1.** CQA reverses lipid dysregulation upon HFD.** Figure S2. **The microbial metabolic behaviors of CQA. **Figure S3.** Gut microbiota play a key role in the anti-obesity effects of CQA. **Figure S4.** Comparison of the biochemical indices between non-responder and responder after chronic CQA treatment. **Figure S5.** *L. reuteri* is susceptible to bacitracin and resistant to vancomycin. **Figure S6.** Intervention of mice with microbial communities lacking or including *L. reuteri* influences the anti-obesity phenotypes of CQA. **Figure S7.** Long-term colonization of *L. reuteri* does not improve the metabolic dysfunctions in DIO mice. **Figure S8.** *L. reuteri* improves metabolic control in DIO mice treated with CQA. **Figure S9.** SCFAs profiling in DIO mice. **Figure S10.** Monocarboxylate transporter is involved in propionate-induced energy expenditure.

**Additional file 2: Supplemental table 1.** Sequences of primers used for quantitative real-time PCR.

## Data Availability

The microbiota raw sequencing data generated in this study has been uploaded to the NCBI SRA database with the accession numbers PRJNA 806994 and PRJNA 904508. The data generated or analyzed during this study are included in this article and its supplementary information files.

## Abbreviations

DIO: Diet-induced obese
CQA: Caffeoylquinic acid
RER: Respiratory exchange ratio
MR: Metabolic rates
WAT: White adipose tissue
BAT: Brown adipose tissue
UCP1: Uncoupling protein 1
Abs: Antibiotics treatment
FMT: Fecal microbiota transplantation
OTU: Operational taxonomic unit
PCA: Principal component analysis
LC-MS: Liquid chromatography-mass spectrometry
SCFAs: Short-chain fatty acids
PA: Propionate
MCT: Monocarboxylic acid transporter
RQ: *L. reuteri* + CQA
