# Correction: *Limosilactobacillus reuteri* and caffeoylquinic acid synergistically promote adipose browning and ameliorate obesity-associated disorders

**DOI:** 10.1186/s40168-023-01641-8

**Published:** 2023-08-15

**Authors:** Yameng Liu, Xianchun Zhong, Suqin Lin, Hualing Xu, Xinyu Liang, Yibin Wang, Jingyi Xu, Kanglong Wang, Xiaozhen Guo, Jiawen Wang, Minjun Yu, Cuina Li, Cen Xie

**Affiliations:** 1 State Key Laboratory of Drug Research, Shanghai Institute of Materia Medica, Chinese Academy of Sciences, Shanghai, 201203 People’s Republic of China; 2 School of Life Sciences, Shanghai University, Shanghai, 200444 People’s Republic of China; 3 School of Chinese Materia Medica, Nanjing University of Chinese Medicine, Nanjing, 210023 People’s Republic of China; 4 University of Chinese Academy of Sciences, Beijing, 100049 People’s Republic of China; 5 Shanghai Key Laboratory of Regulatory Biology, Institute of Biomedical Sciences and School of Life Sciences, East China Normal University, Shanghai, 200241 People’s Republic of China

**Correction: Microbiome 10, 226 (2022)**

**https://doi.org/10.1186/s40168-022-01430-9**

Following publication of the original article [1], the authors reported that Fig. S8e is incorrect. The second and fourth columns were mistakenly the same, as a result of a data pasting error. The authors provided new Fig. S8, and confirmed that this correction has no impact on their findings and conclusions.

The updated additional file is included here and original article has been updated.

### Supplementary Information

**Additional file 1:** **Fig. S8. ***L. reuteri* improves metabolic control in DIO mice treated with CQA. HFD-fed mice were treated twice per week with* L. reuteri *+ CQA (1×10^8^ CFU bacteria, 50 mg/kg CQA) by oral gavage for 5 weeks. Related to Fig. 6. (a) GTT and AUC. (b) Serum HDL-C. (c) Serum LDL-C. (d) Liver weight. (e) Hepatic mRNA expression of lipid synthesis-related genes. (f, g) Representative FL-IR images and BAT temperature. (h) Relative mRNA expression of thermogenic genes in BAT. (i) Representative H&E (upper) and UCP1 (lower) staining of BAT sections, scale bar: 50 μm. *n* = 8/group. Data are presented as mean ± SD. *, *p* < 0.05; **, *p* < 0.01; and ***, *p* < 0.001. ns means not statistically significant.
