# Supplementary material for: Limosilactobacillus reuteri and caffeoylquinic acid synergistically promote adipose browning and ameliorate obesity-associated disorders
Source: Microbiome. 2022 Dec 15;10:226. doi: 10.1186/s40168-022-01430-9 (PMC9753294; doi:10.1186/s40168-022-01430-9)
Supplement: Supplementary file 2 — Additional file 1: Figure S1. CQA reverses lipid dysregulation upon HFD. Figure S2. The microbial metabolic behaviors of CQA. Figure S3. Gut microbiota play a key role in the anti-obesity effects of CQA. Figure S4. Comparison of the biochemical indices between non-responder and responder after chronic CQA treatment. Figure S5. L. reuteri is susceptible to bacitracin and resistant to vancomycin. Figure S6. Intervention of mice with microbial communities lacking or including L. reuteri influences the anti-obesity phenotypes of CQA. Figure S7. Long-term colonization of L. reuteri does not improve the metabolic dysfunctions in DIO mice. Figure S8. L. reuteri improves metabolic control in DIO mice treated with CQA. Figure S9. SCFAs profiling in DIO mice. Figure S10. Monocarboxylate transporter is involved in propionate-induced energy expenditure. [file 40168_2022_1430_MOESM1_ESM.docx]

**Additional file 1**


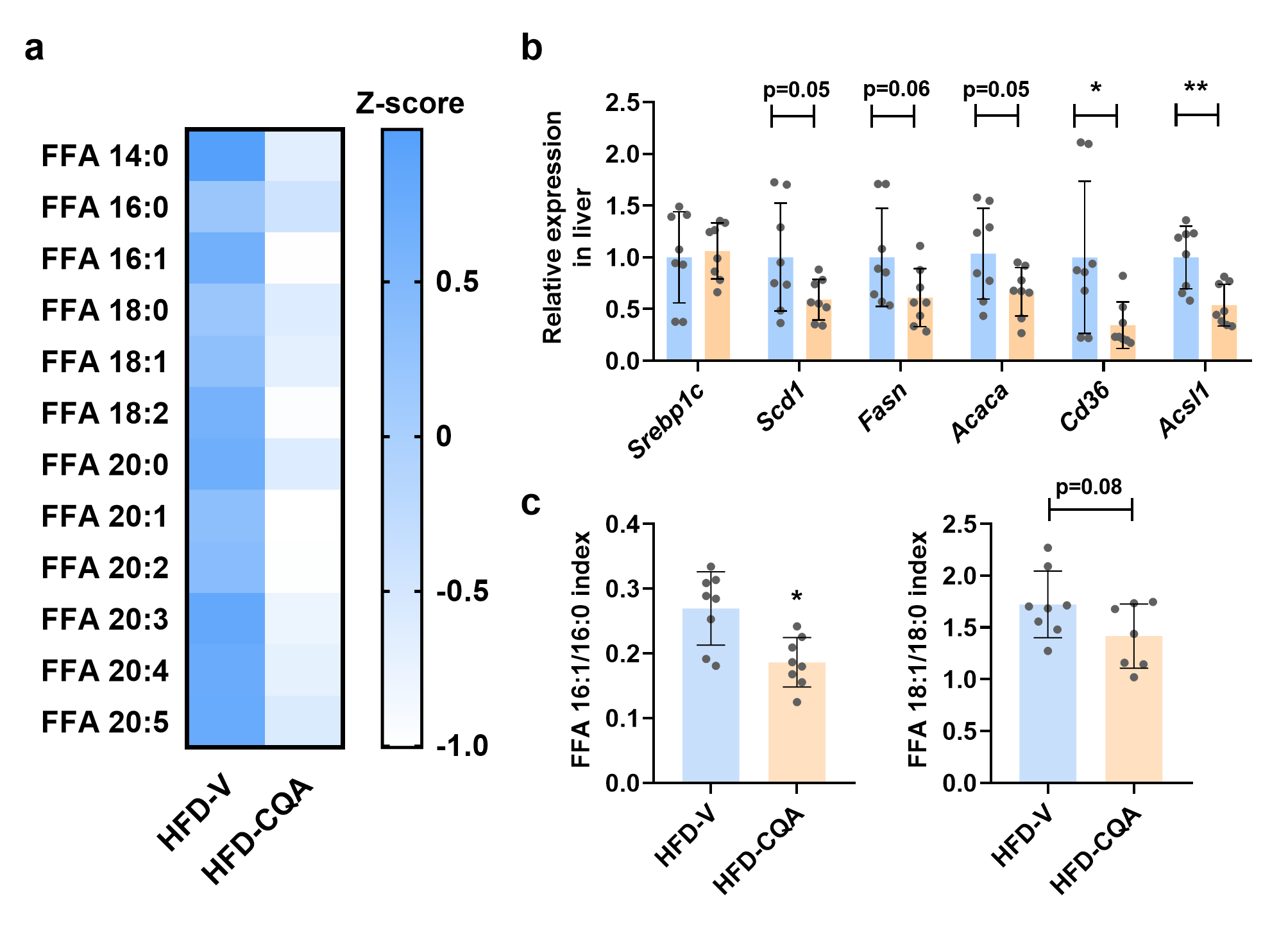


**Fig. S1** CQA reverses lipid dysregulation upon HFD. Related to Fig.1. (a) Serum free fatty acid (FFA) levels. (b) Hepatic mRNA expression of lipid synthesis-related genes. (c) Ratios of FFA16:1/16:0 and 18:1/18:0. n = 7-8/group. Data are presented as mean ± SD. *, p < 0.05; and **, p < 0.01 versus HFD-V.


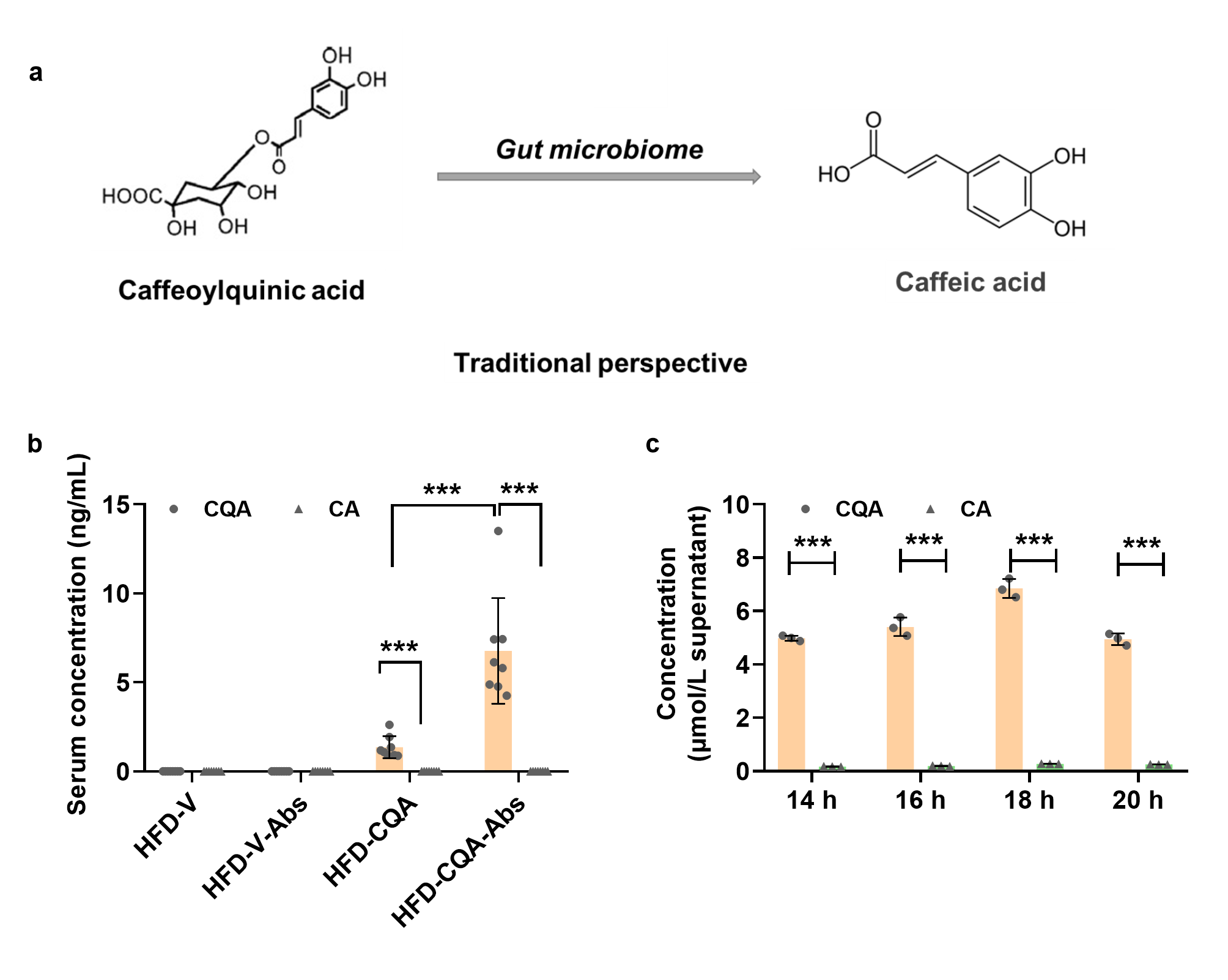


**Fig. S2** The microbial metabolic behaviors of CQA. (a) Schematic diagram of microbial metabolism of CQA from a traditional perspective. (b) Serum concentrations of CQA and its metabolite caffeic acid (CA) in DIO mice (n = 8/group). (c) Concentrations of CQA and CA in the supernatant of *L. reuteri* culture (n = 3/group). Data are presented as mean ± SD. ***, p < 0.001.

**
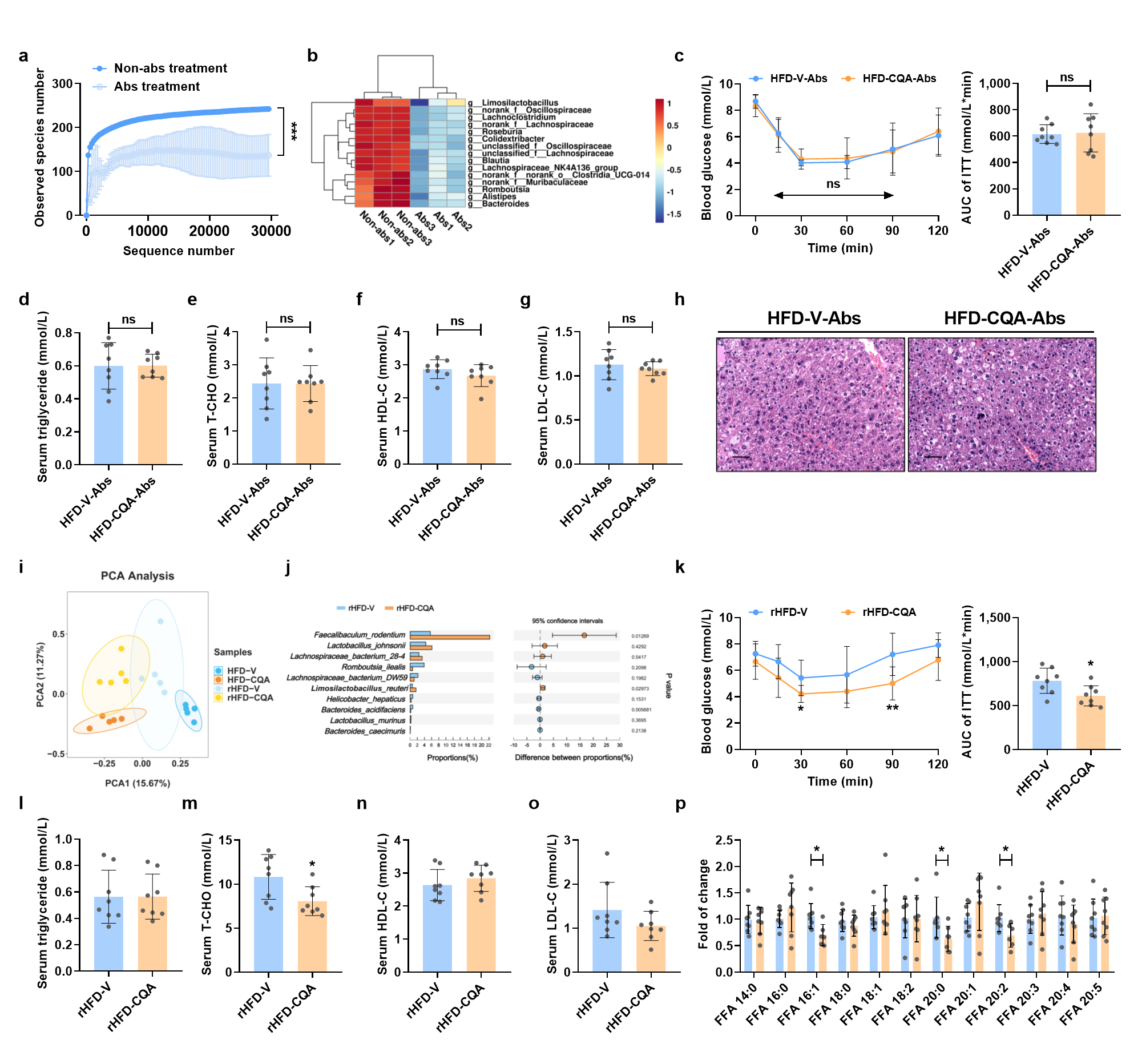
**

**Fig. S3** Gut microbiota play a key role in the anti-obesity effects of CQA. Related to Fig.3. The mice were fed a HFD for 8 weeks and then gavaged with vehicle or CQA (150 mg/kg) together with antibiotics cocktail in drinking water for 8 weeks. (a) Rarefaction curves for 16S rRNA gene amplicons. (b) Heatmap demonstration of bacterial taxa. (c) ITT and AUC of microbiota-reduced mice. (d) Serum triglyceride. (e) Serum T-CHO. (f) Serum HDL-C. (g) Serum LDL-C. (h) Representative H&E staining of liver sections, scale bar: 50 μm. The mice were fed a HFD for 8 weeks and then treated twice per week as recipient mice with fecal microbiota from vehicle-treated or CQA-treated DIO mice for another 8 weeks. (i) Principal component analysis of donors (HFD-V and HFD-CQA group) and recipients (rHFD-V and rHFD-CQA group). (j) Bar plot on species level of recipients (rHFD-V and rHFD-CQA group). (k) ITT and AUC of FMT mice. (l) Serum triglyceride. (m) Serum T-CHO. (n) Serum HDL-C. (o) Serum LDL-C. (p) Serum FFA levels of FMT mice. (a, b) n = 5/group. (c-h) n = 8/group. (i, j) n = 5/group. (k-o) n = 8/group. (p) n = 6-8/group. Data are presented as mean ± SD. *, p < 0.05; and **, p < 0.01; ns means not statistically significant.


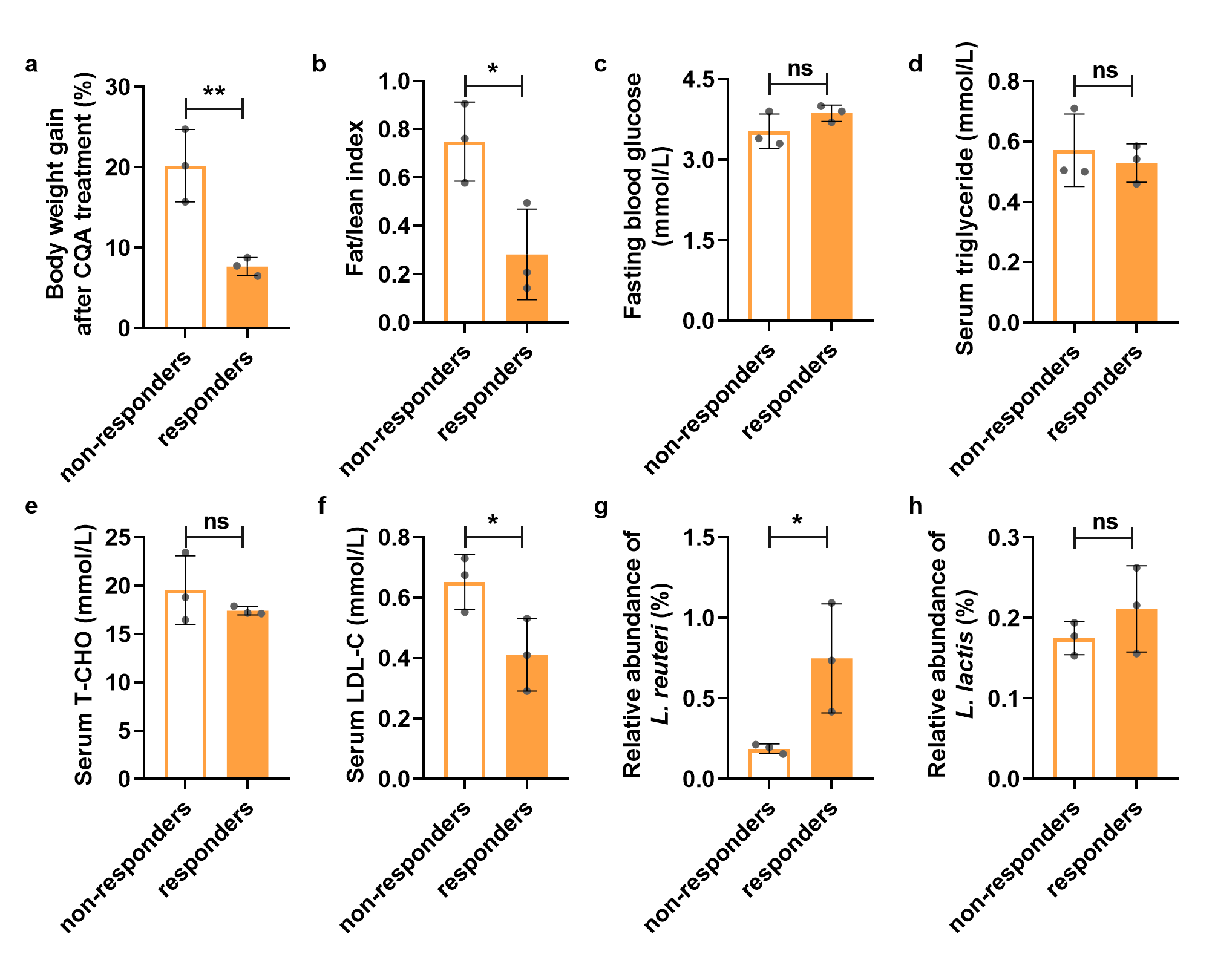


**Fig. S4** Comparison of the biochemical indices between non-responders and responders after long-term CQA treatment. (a) Body weight change. (b) Fat/lean index. (c) Fasting blood glucose. (d) Serum triglyceride. (e) Serum T-CHO. (f) Serum LDL-C. (g) Relative abundance of *L. reuteri* (%). (h) Relative abundance of *L. lactis* (%). n = 3/group. Data are presented as mean ± SD. *, p < 0.05; and **, p < 0.01; ns means not statistically significant.


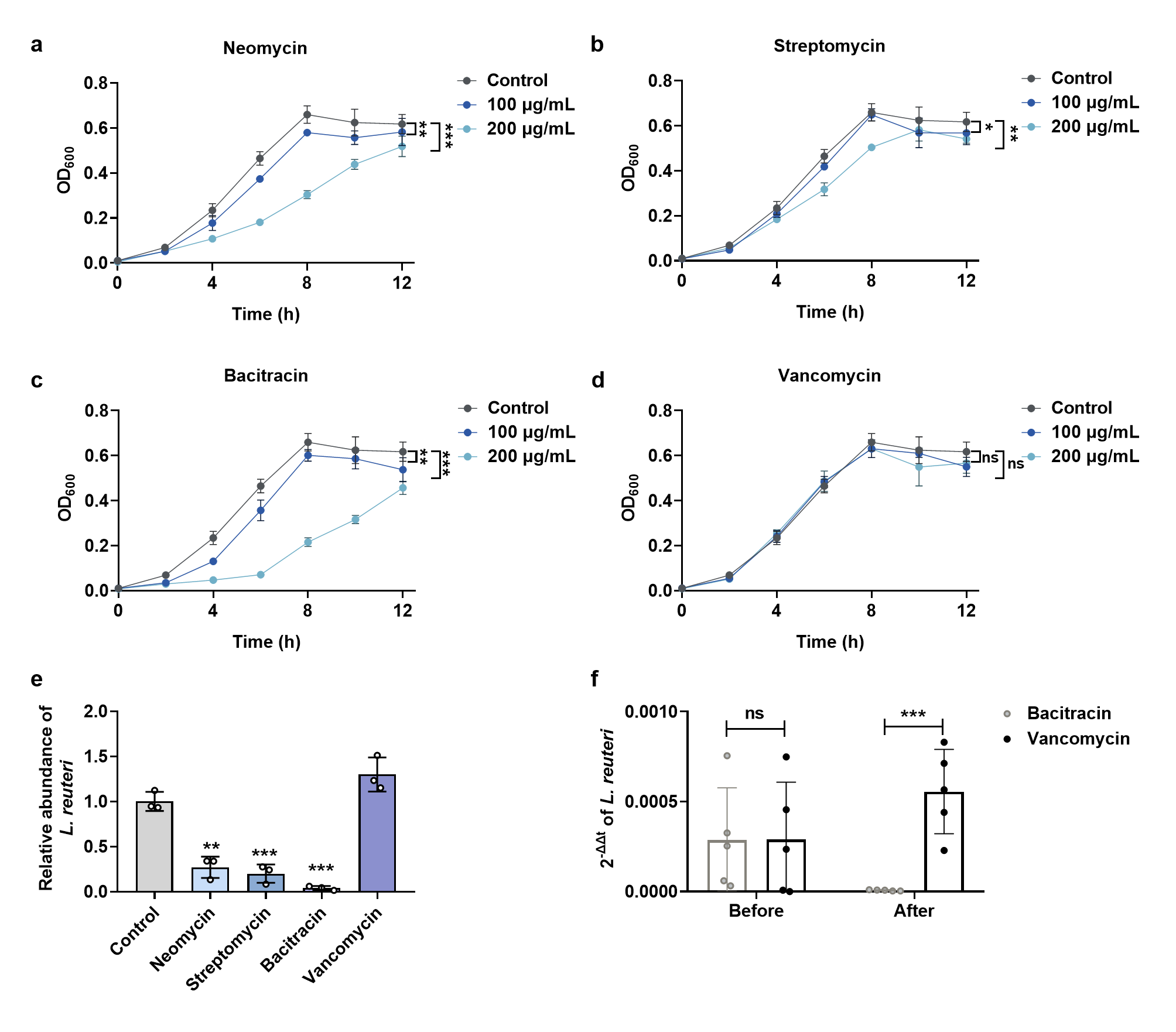


**Fig. S5** *L. reuteri* is susceptible to bacitracin and resistant to vancomycin. Related to Fig.5. (a-d) Growth curves of *L. reuteri* by supplementation with neomycin (a), streptomycin (b), bacitracin (c), and vancomycin (d) in the medium. (e) Relative abundance of *L. reuteri* in anaerobic cultivation of fecal microbiota from DIO mice by supplementation with different antibiotic in the medium. (f) Relative abundance of *L. reuteri* in feces from DIO mice by supplementation with different antibiotic in drinking water. (a-e) n = 3/group. (f) n = 5/group. Data are presented as mean ± SD. *, p < 0.05; **, p < 0.01; and ***, p < 0.001. ns means not statistically significant.


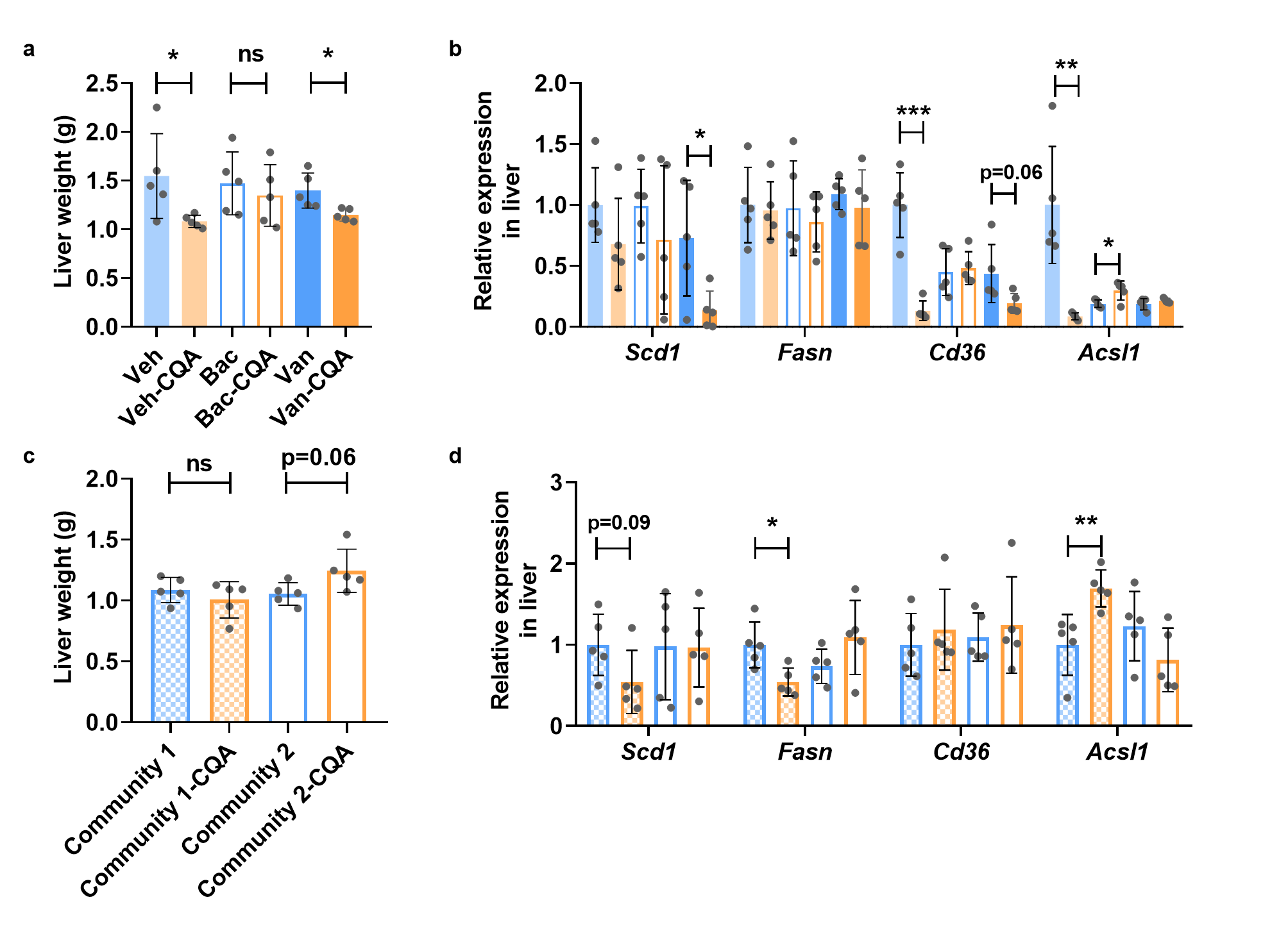


**Fig. S6** Intervention of mice with microbial communities lacking or including *L. reuteri* influences the anti-obesity phenotypes of CQA. Related to Fig.5. The mice were fed a HFD for 10 weeks and then gavaged with vehicle or CQA (150 mg/kg) together with selective antibiotic in drinking water for 4 weeks. (a) Liver weight. (b) Hepatic mRNA expression of lipid synthesis-related genes. The mice were fed a HFD for 10 weeks and then gavaged with vehicle or CQA (150 mg/kg) together with defined microbiota colonization for 4 weeks. (c) Liver weight. (d) Hepatic mRNA expression of lipid synthesis-related genes. n = 5/group. Data are presented as mean ± SD. *, p < 0.05; **, p < 0.01; and ***, p < 0.001. ns means not statistically significant.

**
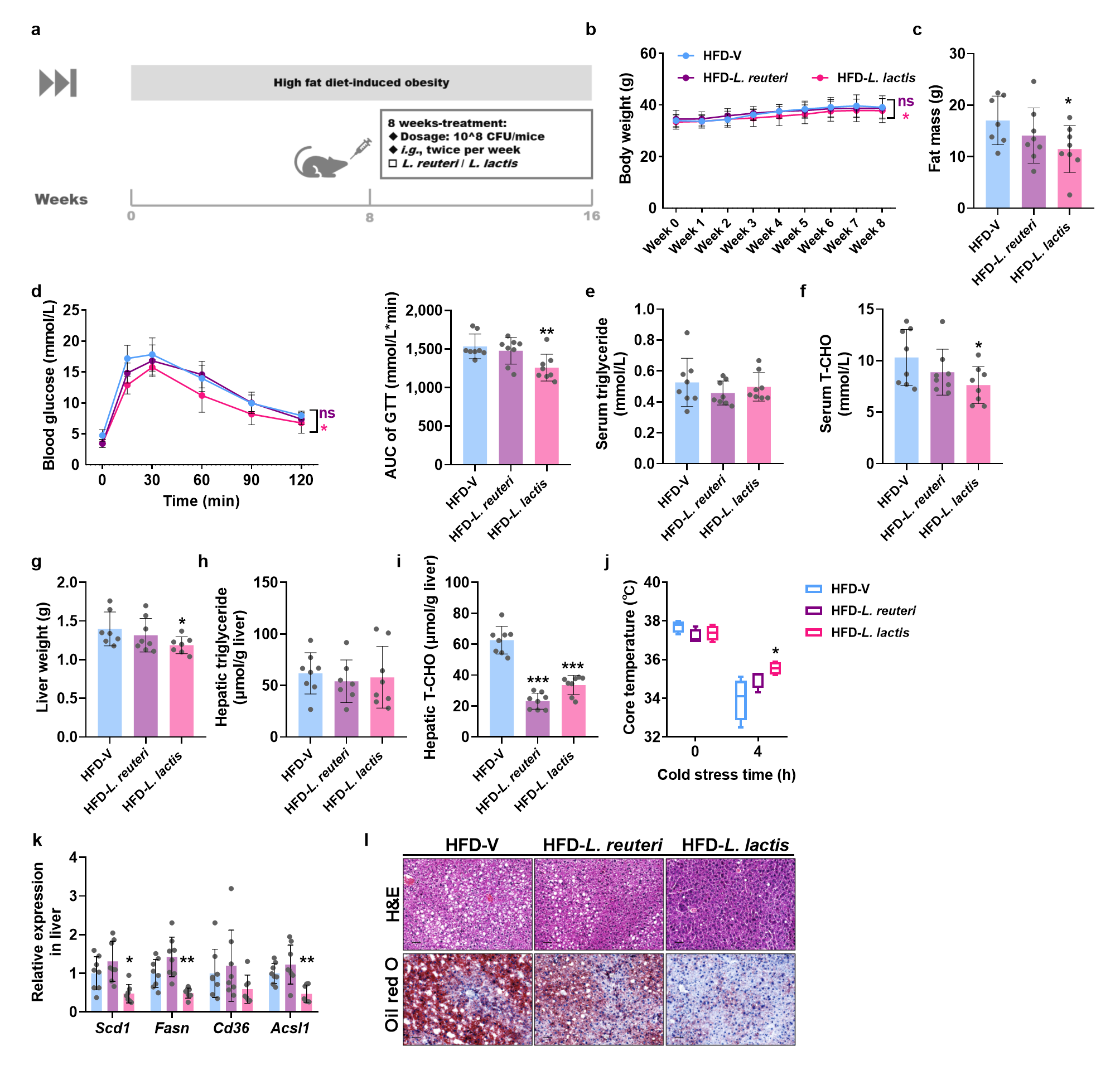
**

**Fig. S7** Long-term treatment of *L. reuteri* does not improve the metabolic dysfunctions in DIO mice. HFD-fed mice were treated twice per week with *L. reuteri* or *L. lactis* (1×10^8^ CFU) by oral gavage for 8 weeks. (a) Schematic diagram of long-term treatment of lactic acid-producing bacteria. (b) Body weight. (c) Fat mass. (d) GTT and AUC. (e) Serum triglyceride. (f) Serum T-CHO. (g) Liver weight. (h) Hepatic triglyceride. (i) Hepatic T-CHO. (j) Core temperature. (k) Hepatic mRNA expression of lipid synthesis-related genes. (l) Representative H&E staining (upper) and Oil red O staining (lower) of liver sections, scale bars: 50 μm. (b-i, k) n = 7-8/group. (j) n = 4/group. Data are presented as mean ± SD. *, p < 0.05; **, p < 0.01; ***, p < 0.001 versus HFD-V. ns means not statistically significant.


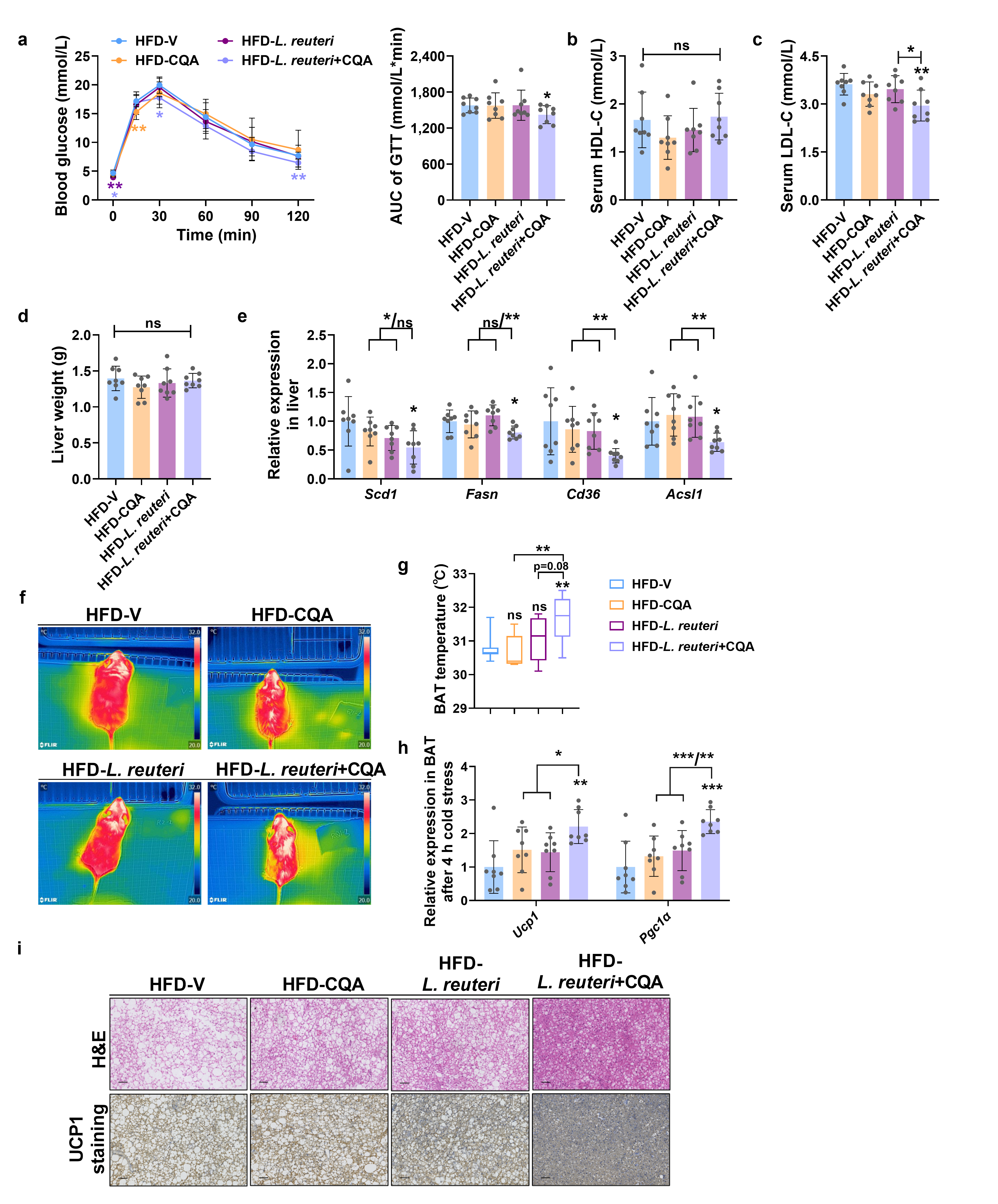


**Fig. S8** *L. reuteri* improves metabolic control in DIO mice treated with CQA. HFD-fed mice were treated twice per week with *L. reuteri* + CQA (1×10^8^ CFU bacteria, 50 mg/kg CQA) by oral gavage for 5 weeks. Related to Fig.6. (a) GTT and AUC. (b) Serum HDL-C. (c) Serum LDL-C. (d) Liver weight. (e) Hepatic mRNA expression of lipid synthesis-related genes. (f, g) Representative FL-IR images and BAT temperature. (h) Relative mRNA expression of thermogenic genes in BAT. (i) Representative H&E (upper) and UCP1 (lower) staining of BAT sections, scale bar: 50 μm. n = 8/group. Data are presented as mean ± SD. *, p < 0.05; **, p < 0.01; and ***, p < 0.001. ns means not statistically significant.


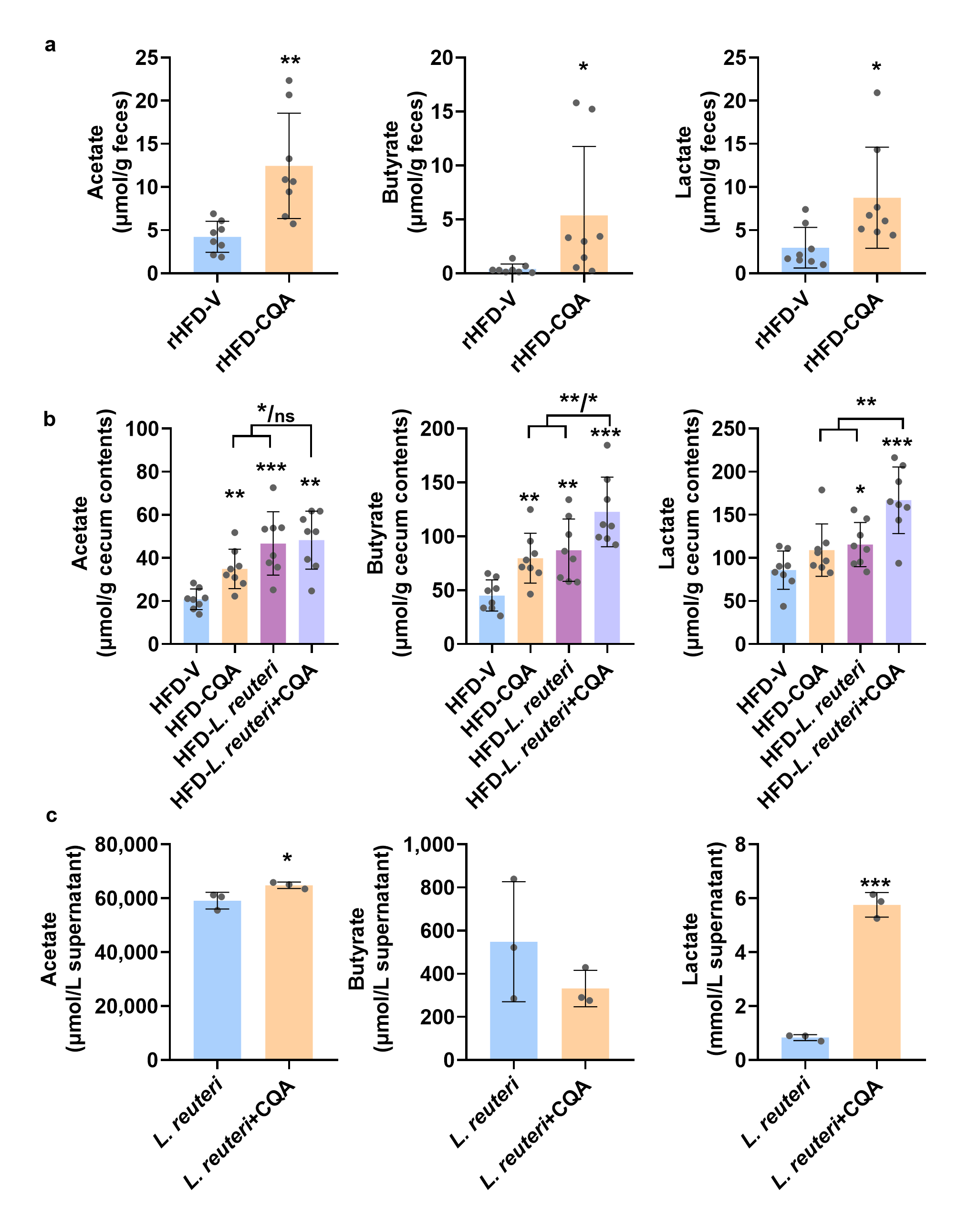


**Fig. S9** SCFAs profiling in DIO mice. Related to Fig.7. (a) Levels of acetate, butyrate, and lactate analyzed in feces after FMT treatment. (b) Levels of acetate, butyrate, and lactate analyzed in cecum contents after *L. reuteri* + CQA co-treatment. (c) Levels of acetate, butyrate, and lactate analyzed in supernatant of *L. reuteri* after co-cultured with CQA. (a, b) n = 8/group. (c) n = 3/group. Data are presented as mean ± SD. *, p < 0.05; **, p < 0.01; ***, p < 0.001. ns means not statistically significant.

**
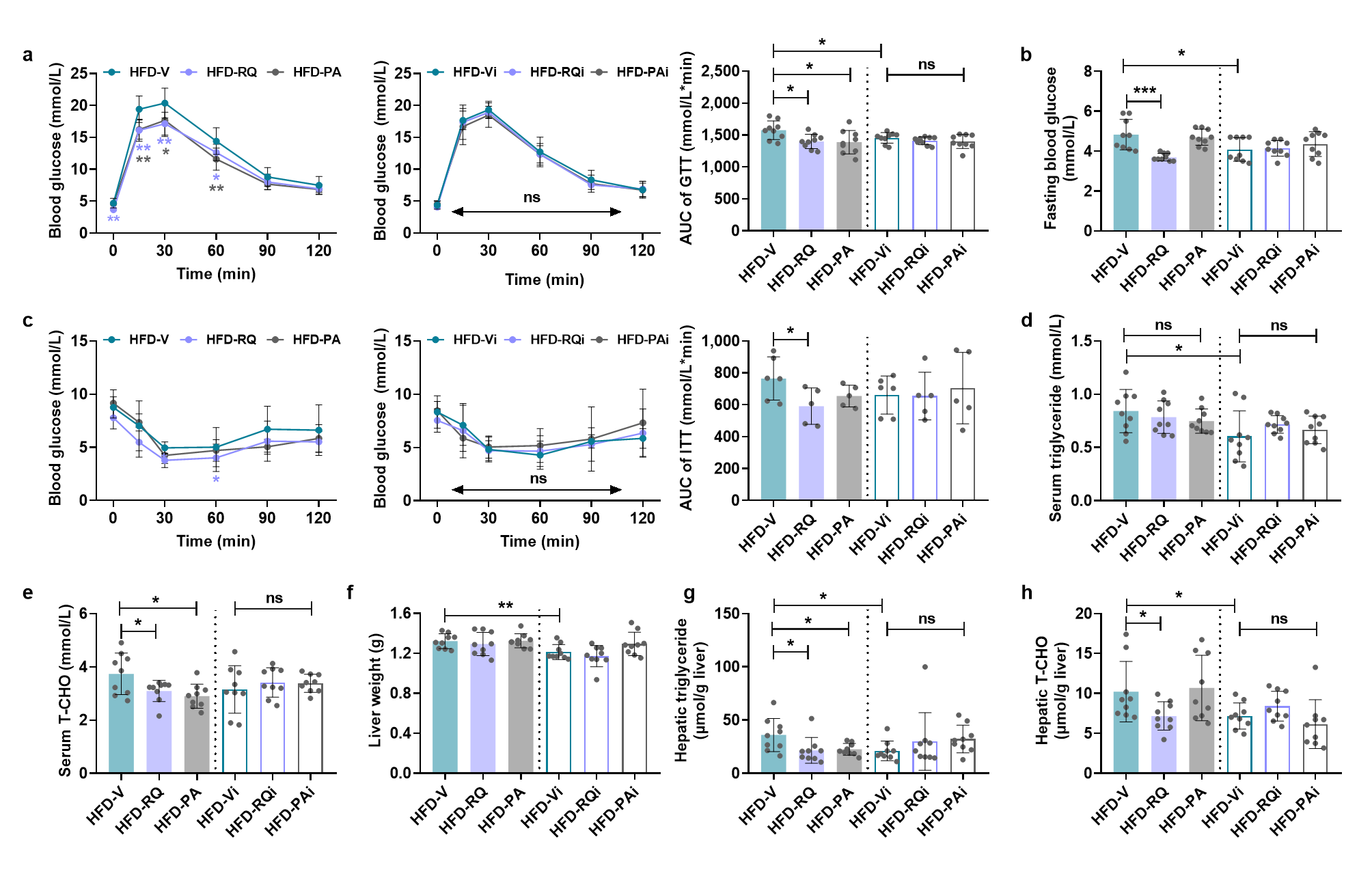
**

**Fig. S10** Monocarboxylate transporter is involved in propionate-induced energy expenditure. Related to Fig.8. (a) GTT and AUC. (b) Fasting blood glucose. (c) ITT and AUC. (d) Serum triglyceride. (e) Serum T-CHO. (f) Liver weight. (g) Hepatic triglyceride. (h) Hepatic T-CHO. (a, b, d-h) n = 9/group. (c) n = 5-6/group. Data are presented as mean ± SD. *, p < 0.05; **, p < 0.01; and ***, p < 0.001. ns means not statistically significant. “RQ” means *L. reuteri* + CQA co-treatment; “PA” means propionate treatment; “i” means treating combined with MCT inhibitor 7ACC1.
