# Supplementary material for: Limosilactobacillus reuteri and caffeoylquinic acid synergistically promote adipose browning and ameliorate obesity-associated disorders
Source: Microbiome. 2022 Dec 15;10:226. doi: 10.1186/s40168-022-01430-9 (PMC9753294; doi:10.1186/s40168-022-01430-9)
Supplement: Supplementary file 3 — Additional file 2: Supplemental table 1. Sequences of primers used for quantitative real-time PCR. [file 40168_2022_1430_MOESM2_ESM.docx]

**Additional file 2**

**Supplemental table 1.** Sequences of primers used for quantitative real-time PCR.

| Mouse primers | Primer sequence |
| --- | --- |
| *Srebp1c* | F:5’-GGAGCCATGGATTGCACATT-3’  R:5’-GCTTCCAGAGAGGAGGCCAG-3’ |
| *Scd1* | F:5’-TTCTTGCGATACACTCTGGTGC-3’  R:5’-CGGGATTGAATGTTCTTGTCGT-3’ |
| *Fasn* | F:5’-AAGTTGCCCGAGTCAGAGAACC-3’  R:5’-ATCCATAGAGCCCAGCCTTCCATC-3’ |
| *Acaca* | F:5’-ATGGGCGGAATGGTCTCTTTC-3’  R:5’-TGGGGACCTTGTCTTCATCAT-3’ |
| *Cd36* | F:5’-AAGCTATTGCGACATGATT-3’  R:5’-GATCCGAACACAGCGTAGAT-3’ |
| *Acsl1* | F:5’-CGATGGCTGTTGGACTTTGC-3’  R:5’-CACCCAGGCTCGACTGTATC-3’ |
| *Ucp1* | F:5’-AGGCTTCCAGTACCATTAGGT-3’  R:5’-CTGAGTGAGGCAAAGCTGATTT-3’ |
| *Pgc1a* | F:5’-TATGGAGTGACATAGAGTGTGCT-3’  R:5’-CCACTTCAATCCACCCAGAAAG-3’ |
| *Actin* | F:5’-GGCTGTATTCCCCTCCATCG-3’  R:5’-CCAGTTGGTAACAATGCCATGT-3’ |
| *Gapdh* | F:5’-AGGTCGGTGTGAACGGATTTG-3’  R:5’-TGTAGACCATGTAGTTGAGGTCA-3’ |
| *18s* | F:5’-ATTGGAGCTGGAATTACCGC-3’  R:5’-CGGCTACCACATCCAAGGAA-3’ |

| Microbe primers | Primer sequence |
| --- | --- |
| *Lactococcus lactis* | F: 5’-GAGTAACGCGTGGGGAATCT-3’  R: 5’-CTGCCTCCCGTAGGAGTTTG-3’ |
| *Limosilactobacillus reuteri* | F: 5’-GTGCTTGCACCTGATTGACG-3’  R: 5’-CCATTGTGGCCGATCAGTCT-3’ |
| *univ63_335* | F: 5’-GCAGGCCTAACACATGCAAGTC-3’  R: 5’-CTGCTGCCTCCCGTAGGAGT-3’ |
| *univ338_518* | F: 5’-ACTCCTACGGGAGGCAGCAG-3’  R: 5’-ATTACCGCGGCTGCTGG-3’ |
| *univ907_1062* | F: 5’-AAACTCAAAKGAATTGACGG-3’  R: 5’-CTCACRRCACGAGCTGAC-3’ |
