# Supplementary material for: Correction: Limosilactobacillus reuteri and caffeoylquinic acid synergistically promote adipose browning and ameliorate obesity-associated disorders
Source: Microbiome. 2023 Aug 15;11:182. doi: 10.1186/s40168-023-01641-8 (PMC10426170; doi:10.1186/s40168-023-01641-8)
Supplement: Supplementary file 1 — Additional file 1: Fig. S8. L. reuteri improves metabolic control in DIO mice treated with CQA. HFD-fed mice were treated twice per week with L. reuteri + CQA (1×10^8^ CFU bacteria, 50 mg/kg CQA) by oral gavage for 5 weeks. Related to Fig. 6. (a) GTT and AUC. (b) Serum HDL-C. (c) Serum LDL-C. (d) Liver weight. (e) Hepatic mRNA expression of lipid synthesis-related genes. (f, g) Representative FL-IR images and BAT temperature. (h) Relative mRNA expression of thermogenic genes in BAT. (i) Representative H&E (upper) and UCP1 (lower) staining of BAT sections, scale bar: 50 μm. n = 8/group. Data are presented as mean ± SD. *, p < 0.05; **, p < 0.01; and ***, p < 0.001. ns means not statistically significant. [file 40168_2023_1641_MOESM1_ESM.docx]

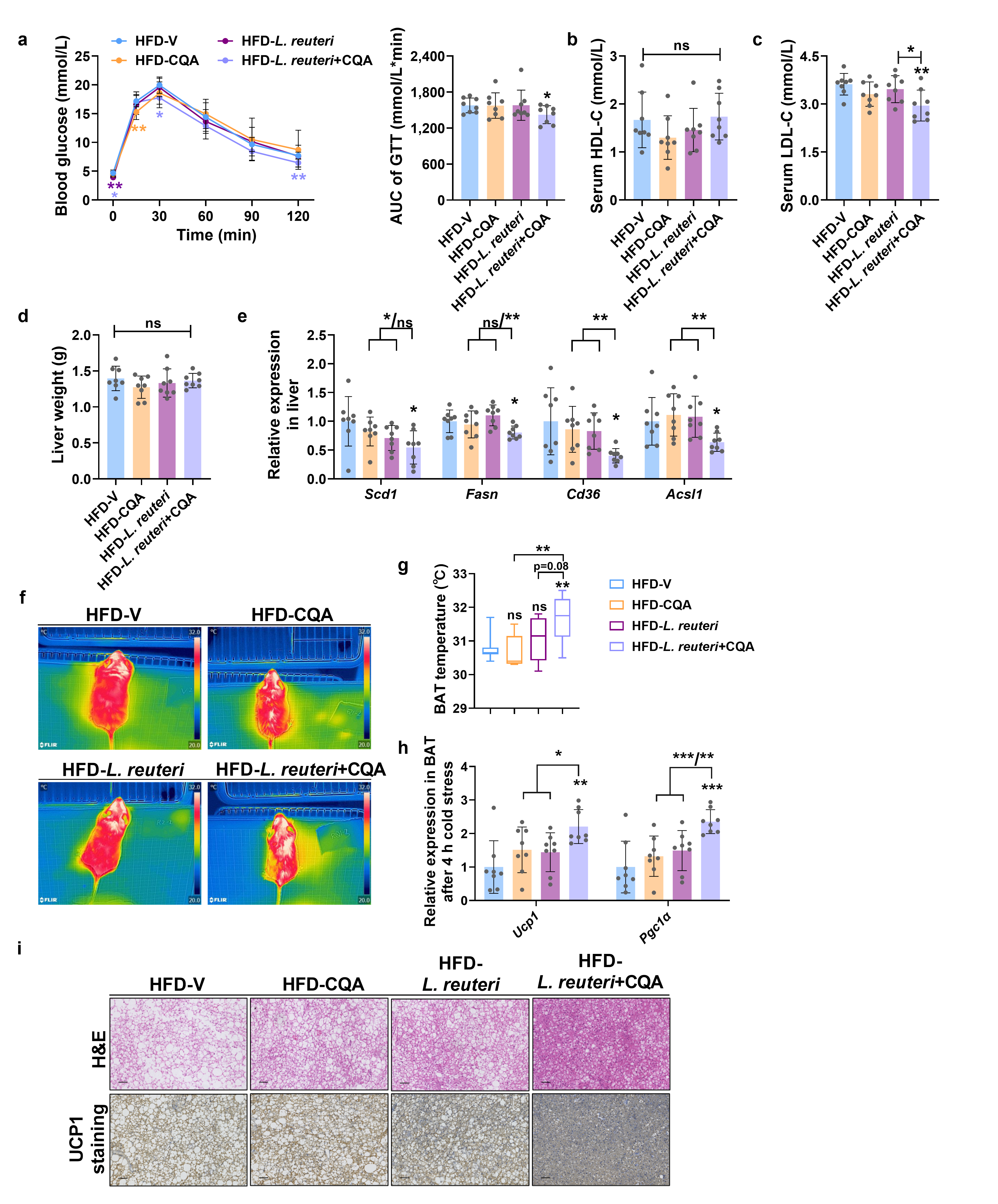


**Fig. S8** *L. reuteri* improves metabolic control in DIO mice treated with CQA. HFD-fed mice were treated twice per week with *L. reuteri* + CQA (1×10^8^ CFU bacteria, 50 mg/kg CQA) by oral gavage for 5 weeks. Related to Fig.6. (a) GTT and AUC. (b) Serum HDL-C. (c) Serum LDL-C. (d) Liver weight. (e) Hepatic mRNA expression of lipid synthesis-related genes. (f, g) Representative FL-IR images and BAT temperature. (h) Relative mRNA expression of thermogenic genes in BAT. (i) Representative H&E (upper) and UCP1 (lower) staining of BAT sections, scale bar: 50 μm. n = 8/group. Data are presented as mean ± SD. *, p < 0.05; **, p < 0.01; and ***, p < 0.001. ns means not statistically significant.
